# Supplementary figures and images for: Repeatability and timing of tropical influenza epidemics
Source: PLoS Comput Biol. 2023 Jul 19;19(7):e1011317. doi: 10.1371/journal.pcbi.1011317 (PMC10389745; doi:10.1371/journal.pcbi.1011317)

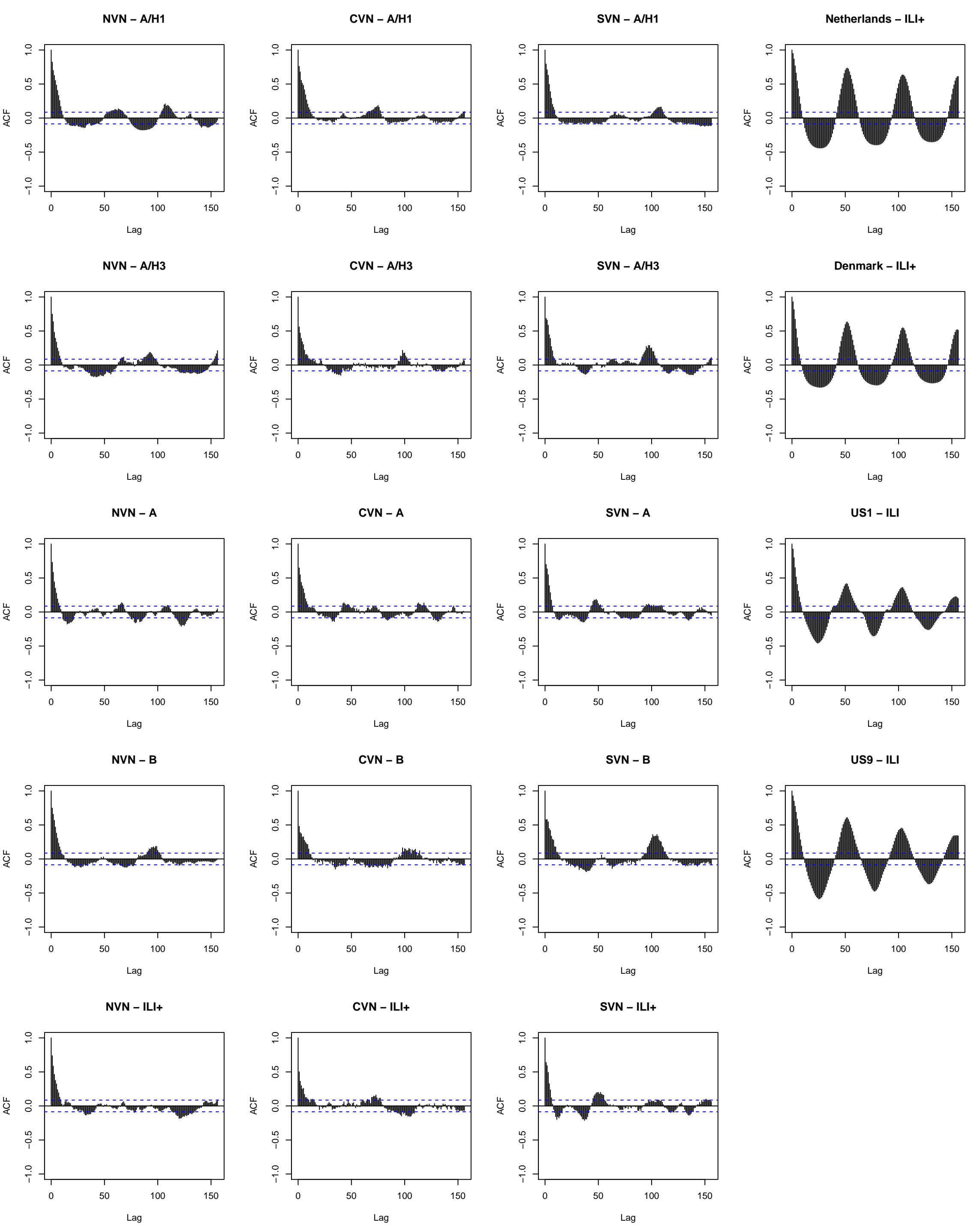

Supplement: S1 Fig — Columns from left to right show autocorrelation plots for northern Vietnam, central Vietnam, southern Vietnam, and four temperate locations. (PDF) [file pcbi.1011317.s001.pdf]

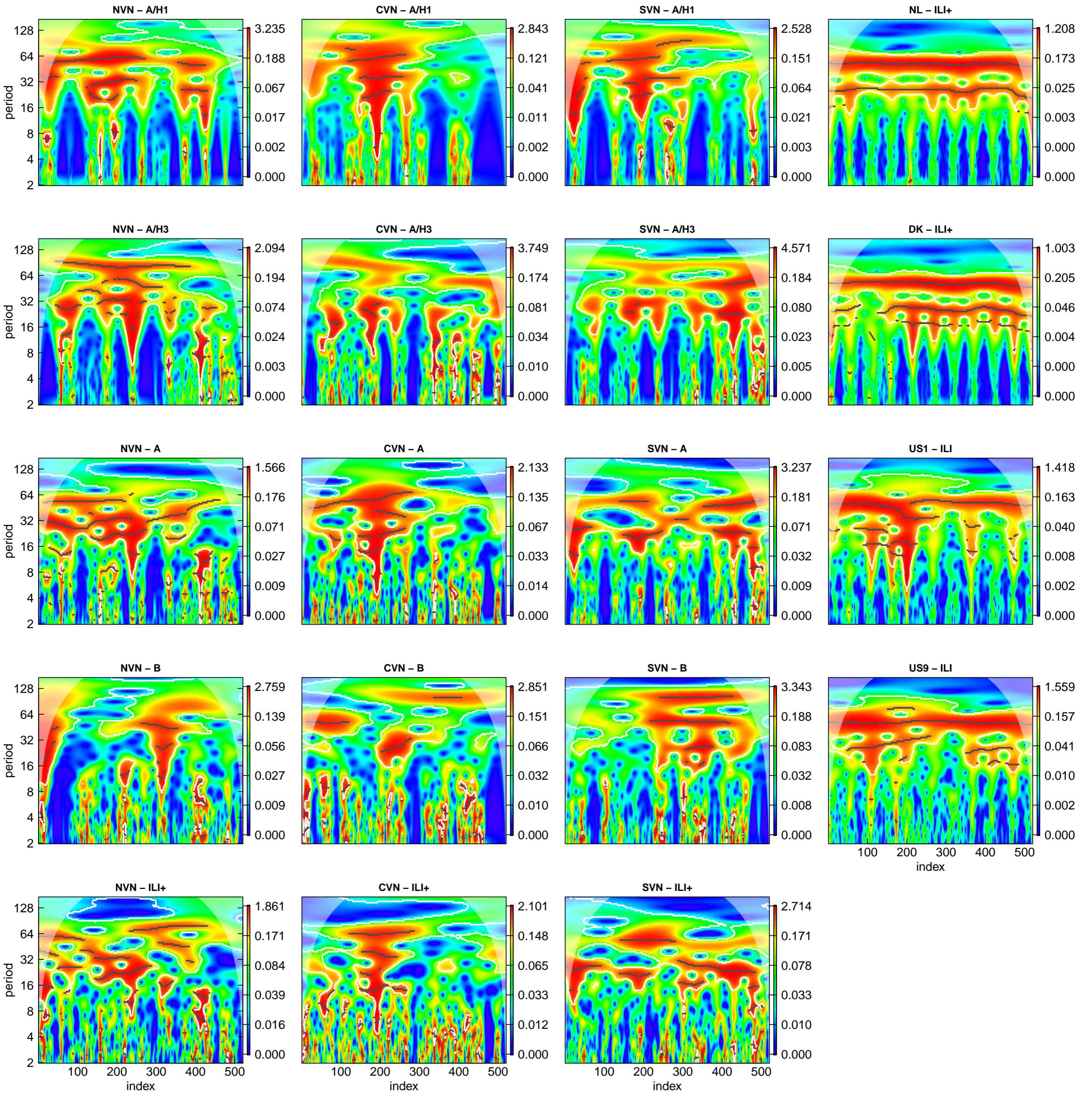

Supplement: S2 Fig — Columns from left to right show results for northern Vietnam, central Vietnam, southern Vietnam, and temperate locations. (PDF) [file pcbi.1011317.s002.pdf]

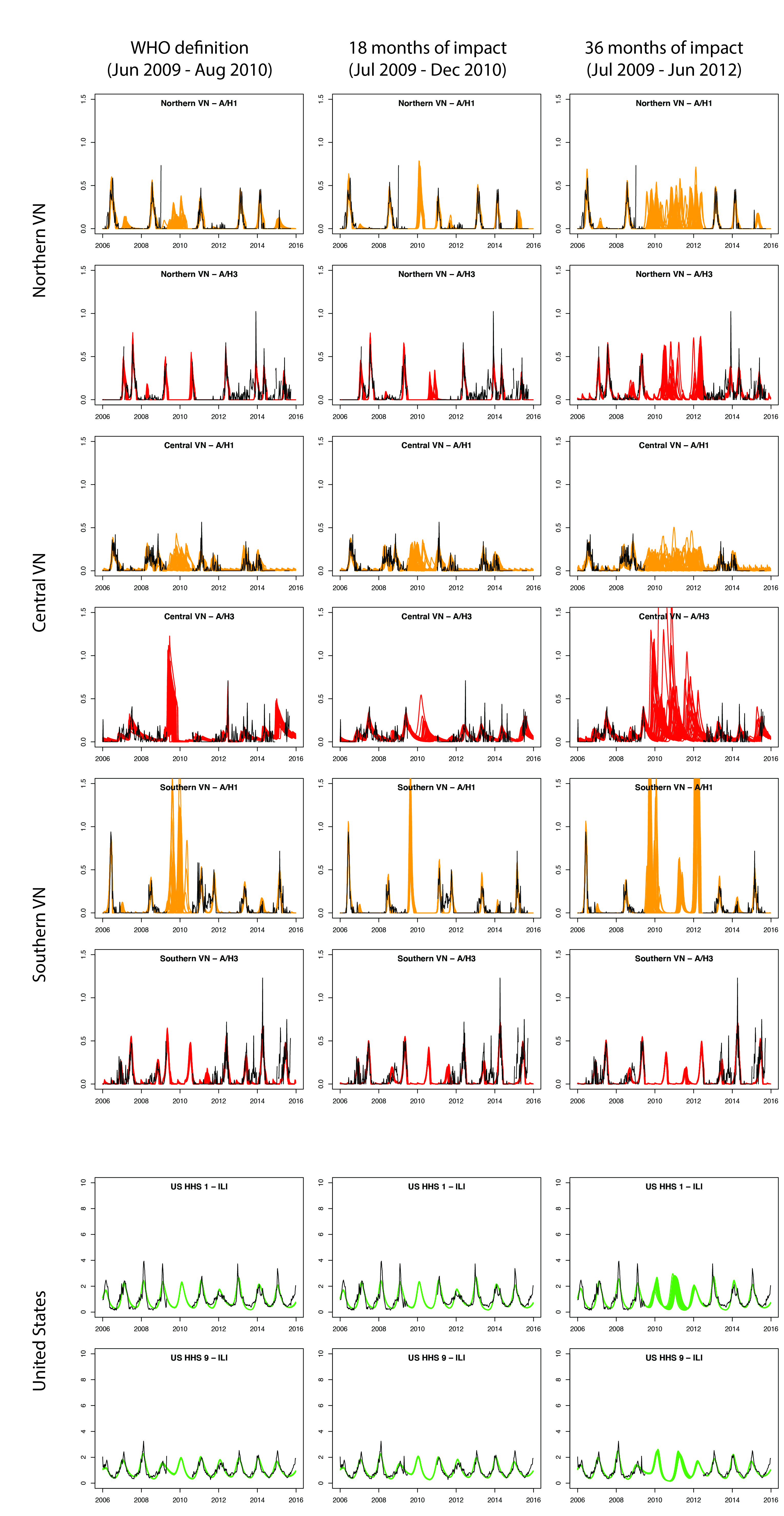

Supplement: S4 Fig — Omitted times include the WHO definition of June 2009 –August 2010 (left column); 18 months from the start of the pandemic’s influenza on Vietnam, July 2009 –December 2010 (middle column); and 36 months from the start of the pandemic’s influence on Vietnam, July 2009 –June 2012 (right column). (JPG) [file pcbi.1011317.s004.jpg]

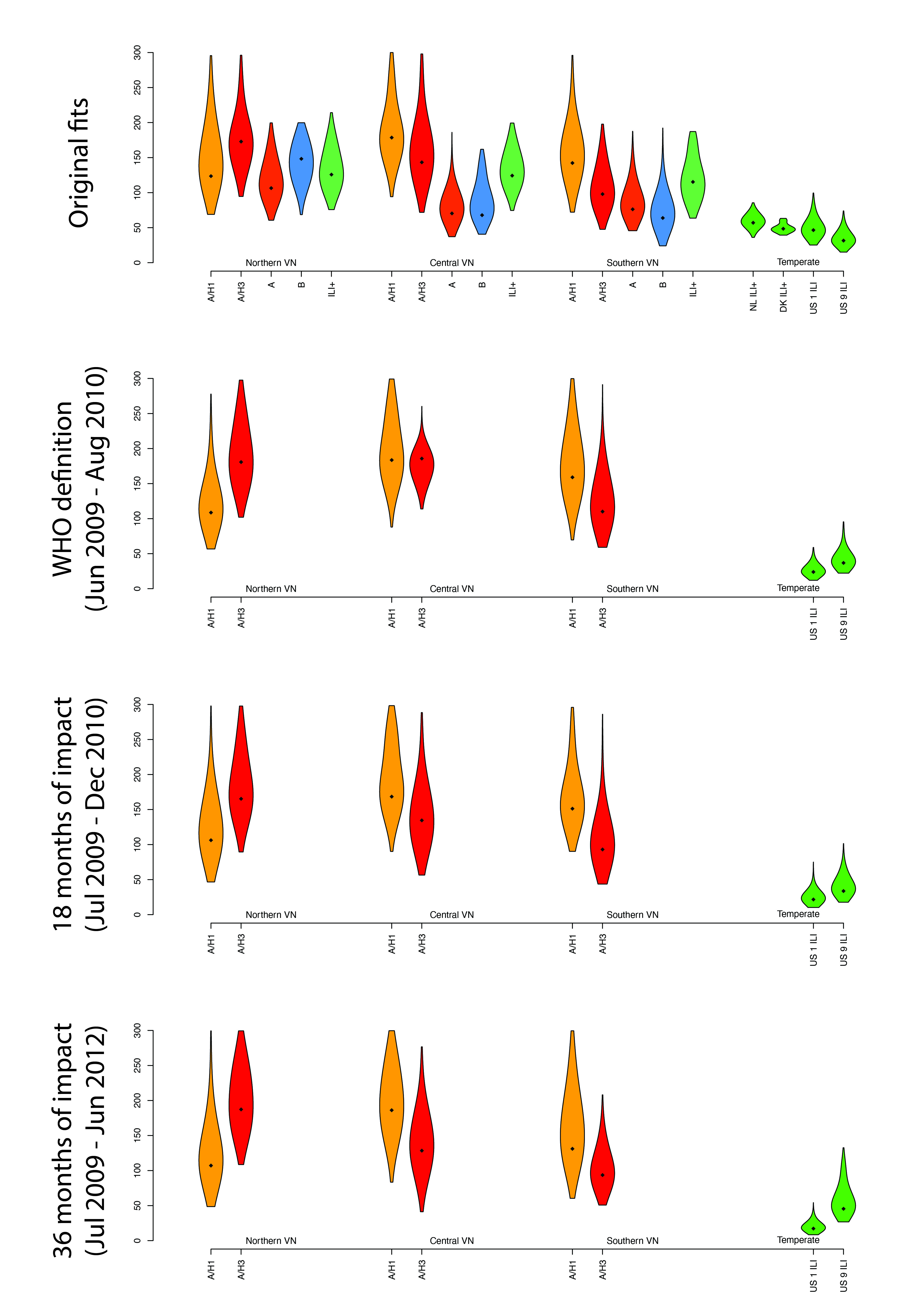

Supplement: S5 Fig — The original fits are shown in the top panel. Omitted times include the WHO definition of June 2009 –August 2010 (second panel); 18 months from the start of the pandemic’s influence on Vietnam, July 2009 –December 2010 (third panel); and 36 months from the start of the pandemic’s influence on Vietnam, July 2009 –June 2012 (fourth panel). (JPG) [file pcbi.1011317.s005.jpg]
