# Supplementary material for: Repeatability and timing of tropical influenza epidemics
Source: PLoS Comput Biol. 2023 Jul 19;19(7):e1011317. doi: 10.1371/journal.pcbi.1011317 (PMC10389745; doi:10.1371/journal.pcbi.1011317)
Supplement: S3 Fig — Individual model fits are shown with median values denoted by grey points and mode values denoted by red points. Locations and influenza (sub)types are indicated on the top left corner of each page. The location codes are: nvn–northern Vietnam, cvn–central Vietnam, svn–southern Vietnam, nl–Netherlands, dk–Denmark, us1 –United States region 1, us9 –United States region 9. The (sub)type codes are: h1 –subtype A/H1, h3 –subtype A/H3, a–type A, b–type B, c–combined ILI+. (PDF) [file pcbi.1011317.s003.pdf]

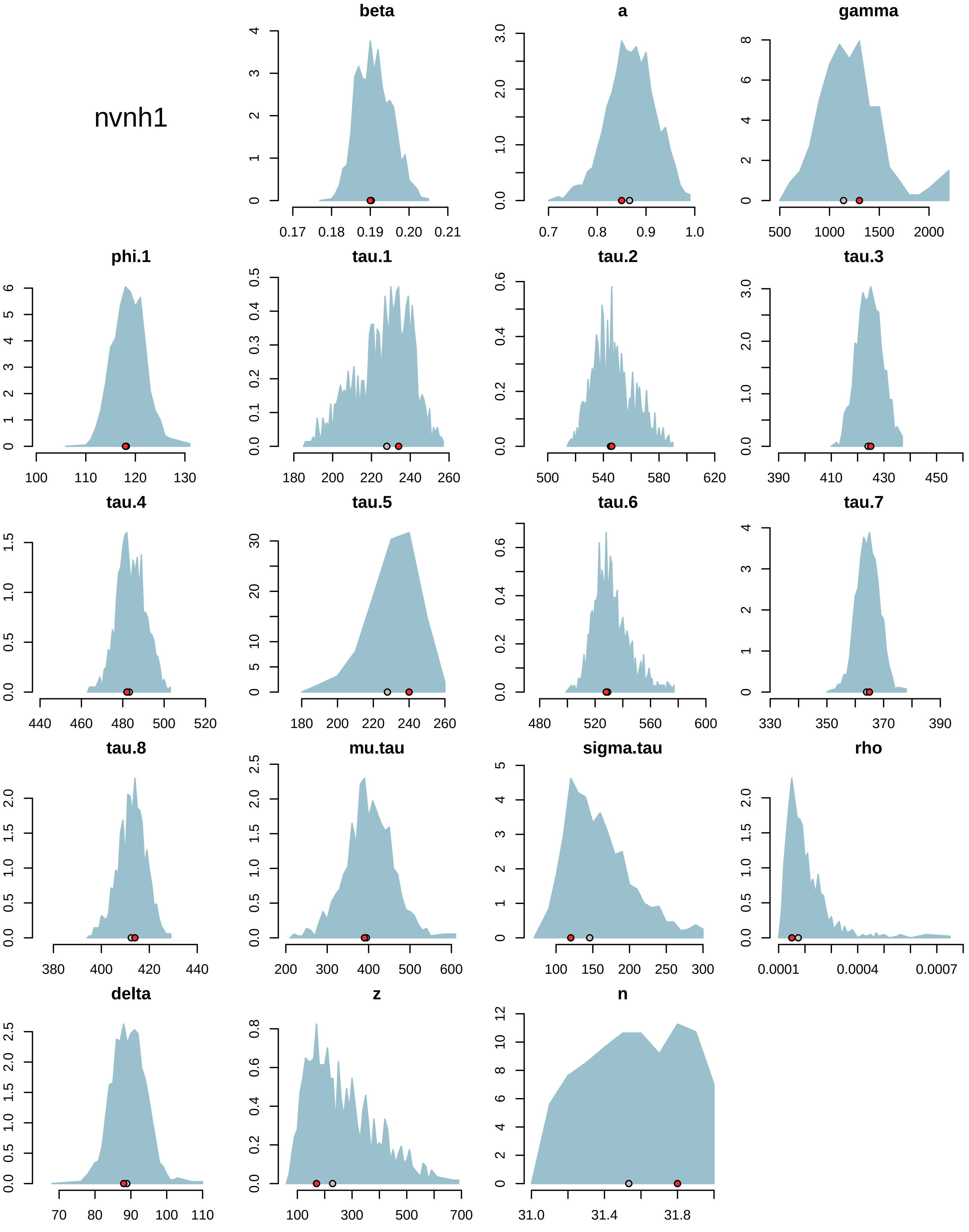
nvnh1
beta
0.17 0.18 0.19 0.20 0.21
a
0.7 0.8 0.9 1.0
gamma
500 1000 1500 2000
phi.1
100 110 120 130
tau.1
180 200 220 240 260
tau.2
500 540 580 620
tau.3
390 410 430 450
tau.4
440 460 480 500 520
tau.5
180 200 220 240 260
tau.6
480 520 560 600
tau.7
330 350 370 390
tau.8
380 400 420 440
mu.tau
200 300 400 500 600
sigma.tau
100 150 200 250 300
rho
0.0001 0.0004 0.0007
delta
70 80 90 100 110
z
100 300 500 700
n
31.0 31.4 31.8

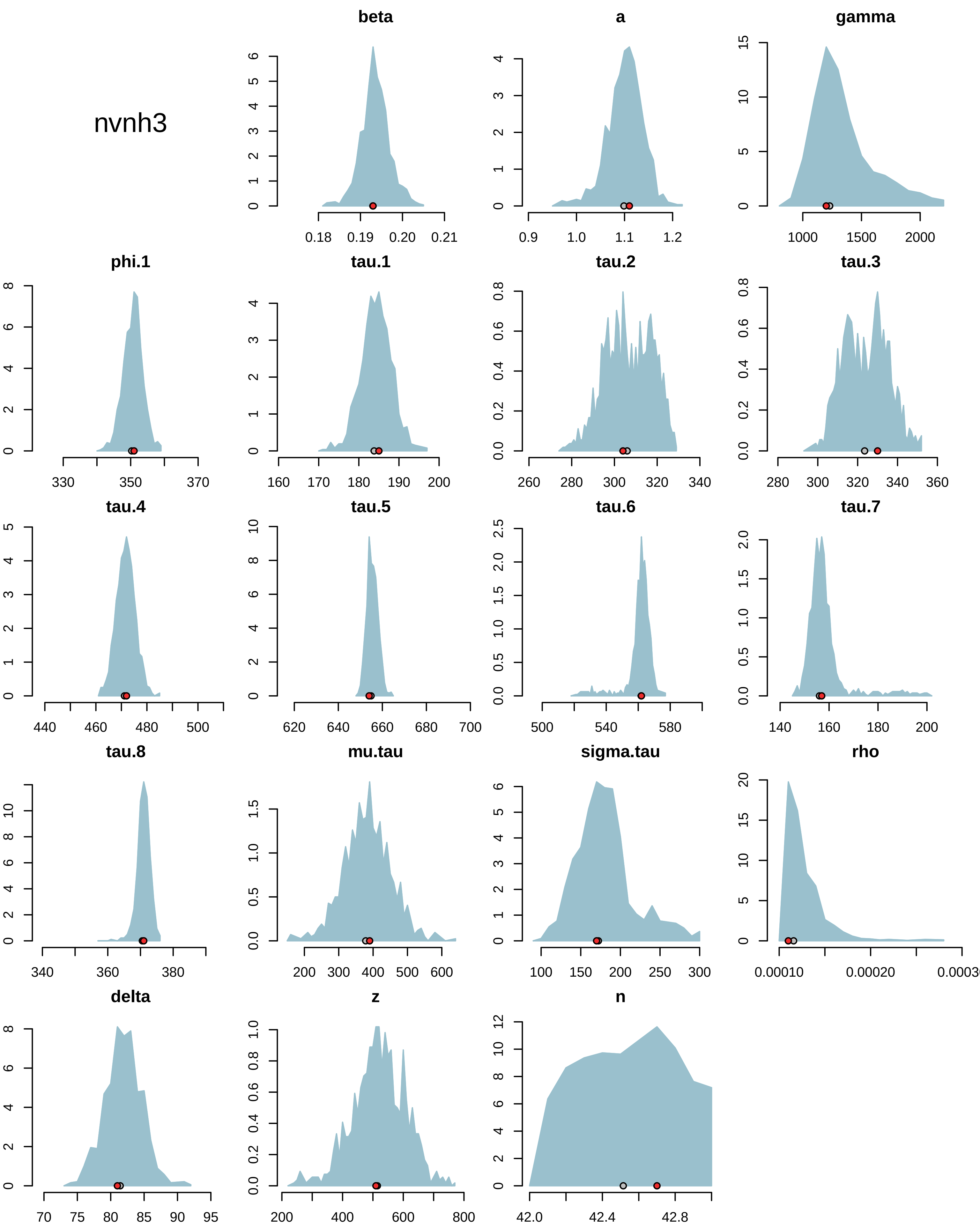
nvnh3
beta
0.18 0.19 0.20 0.21
a
0.9 1.0 1.1 1.2
gamma
1000 1500 2000
phi.1
330 350 370
tau.1
160 170 180 190 200
tau.2
260 280 300 320 340
tau.3
280 300 320 340 360
tau.4
440 460 480 500
tau.5
620 640 660 680 700
tau.6
500 540 580
tau.7
140 160 180 200
tau.8
340 360 380
mu.tau
200 300 400 500 600
sigma.tau
100 150 200 250 300
rho
0.00010 0.00020
delta
70 75 80 85 90 95
z
200 400 600 800
n
42.0 42.4 42.8

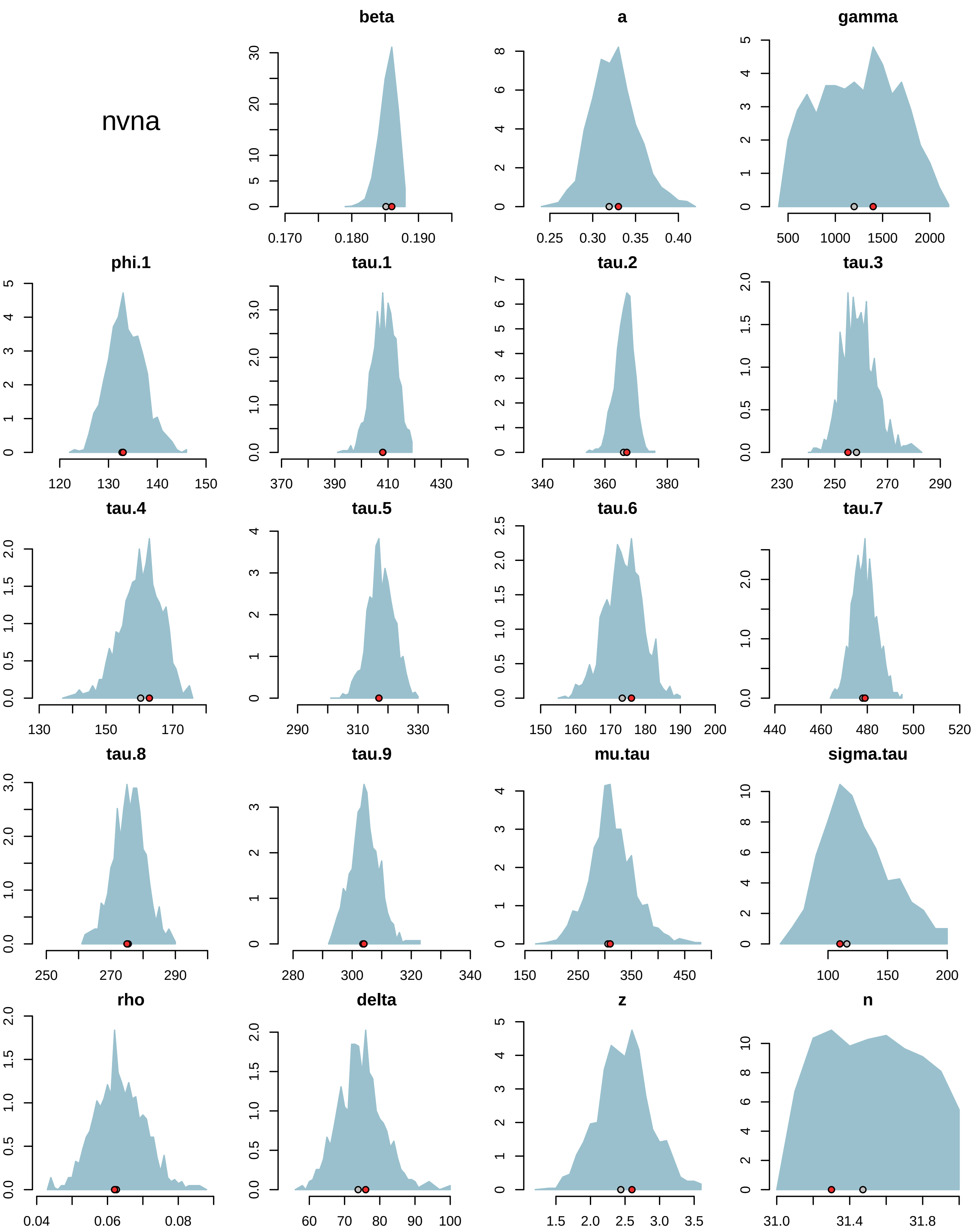
nvna
beta
0 5 10 20 30
0.170 0.180 0.190
a
0 2 4 6 8
0.25 0.30 0.35 0.40
gamma
0 1 2 3 4 5
500 1000 1500 2000
phi.1
0 1 2 3 4 5
120 130 140 150
tau.1
0.0 1.0 2.0 3.0
370 390 410 430
tau.2
0 1 2 3 4 5 6 7
340 360 380
tau.3
0.0 0.5 1.0 1.5 2.0
230 250 270 290
tau.4
0.0 0.5 1.0 1.5 2.0
130 150 170
tau.5
0 1 2 3 4
290 310 330
tau.6
0.0 0.5 1.0 1.5 2.0 2.5
150 160 170 180 190 200
tau.7
0.0 1.0 2.0
440 460 480 500 520
tau.8
0.0 1.0 2.0 3.0
250 270 290
tau.9
0 1 2 3
280 300 320 340
mu.tau
0 1 2 3 4
150 250 350 450
sigma.tau
0 2 4 6 8 10
100 150 200
rho
0.0 0.5 1.0 1.5 2.0
0.04 0.06 0.08
delta
0.0 0.5 1.0 1.5 2.0
60 70 80 90 100
z
0 1 2 3 4 5
1.5 2.0 2.5 3.0 3.5
n
0 2 4 6 8 10
31.0 31.4 31.8

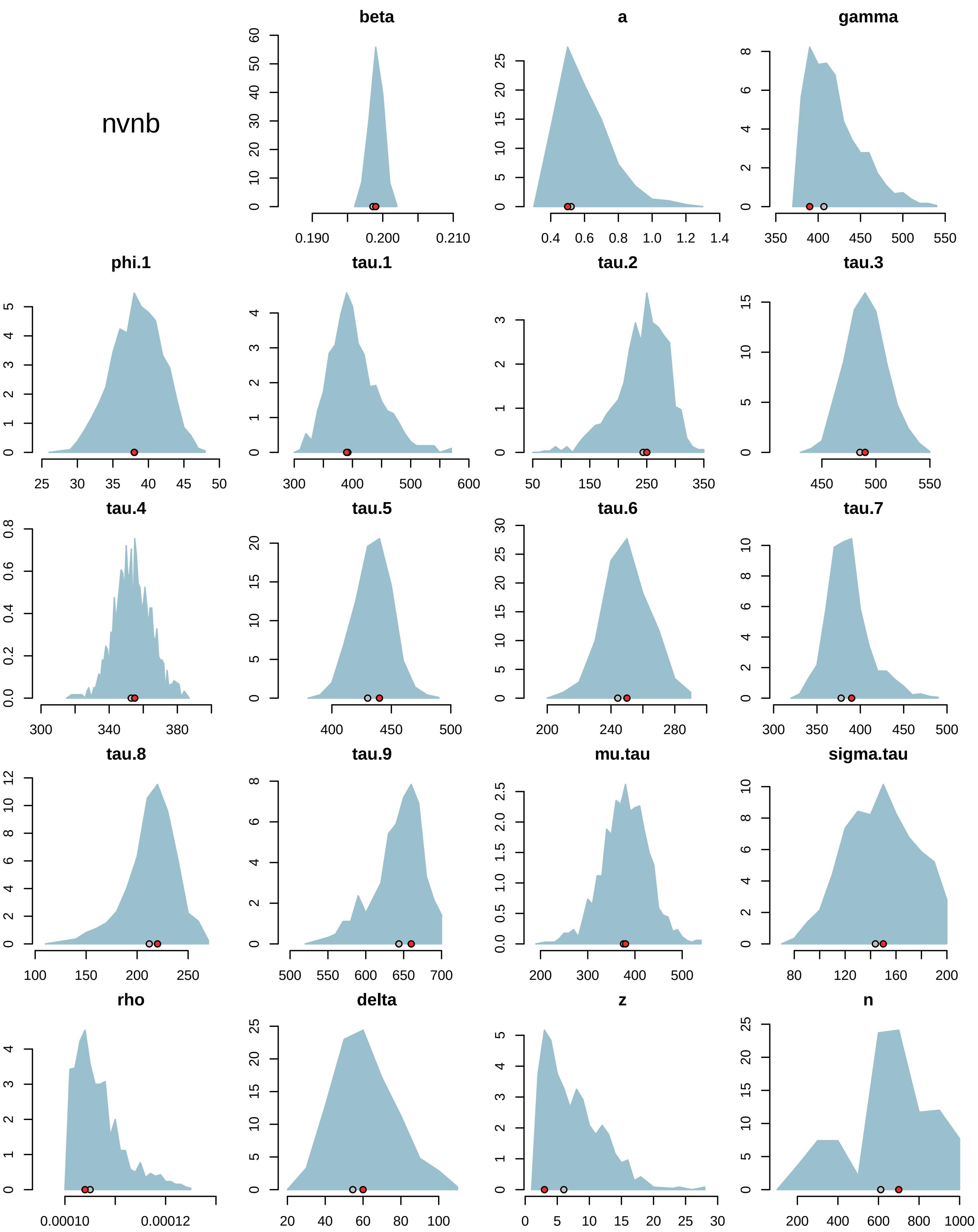
nvnb
beta
a
gamma
phi.1
tau.1
tau.2
tau.3
tau.4
tau.5
tau.6
tau.7
tau.8
tau.9
mu.tau
sigma.tau
rho
delta
z
n

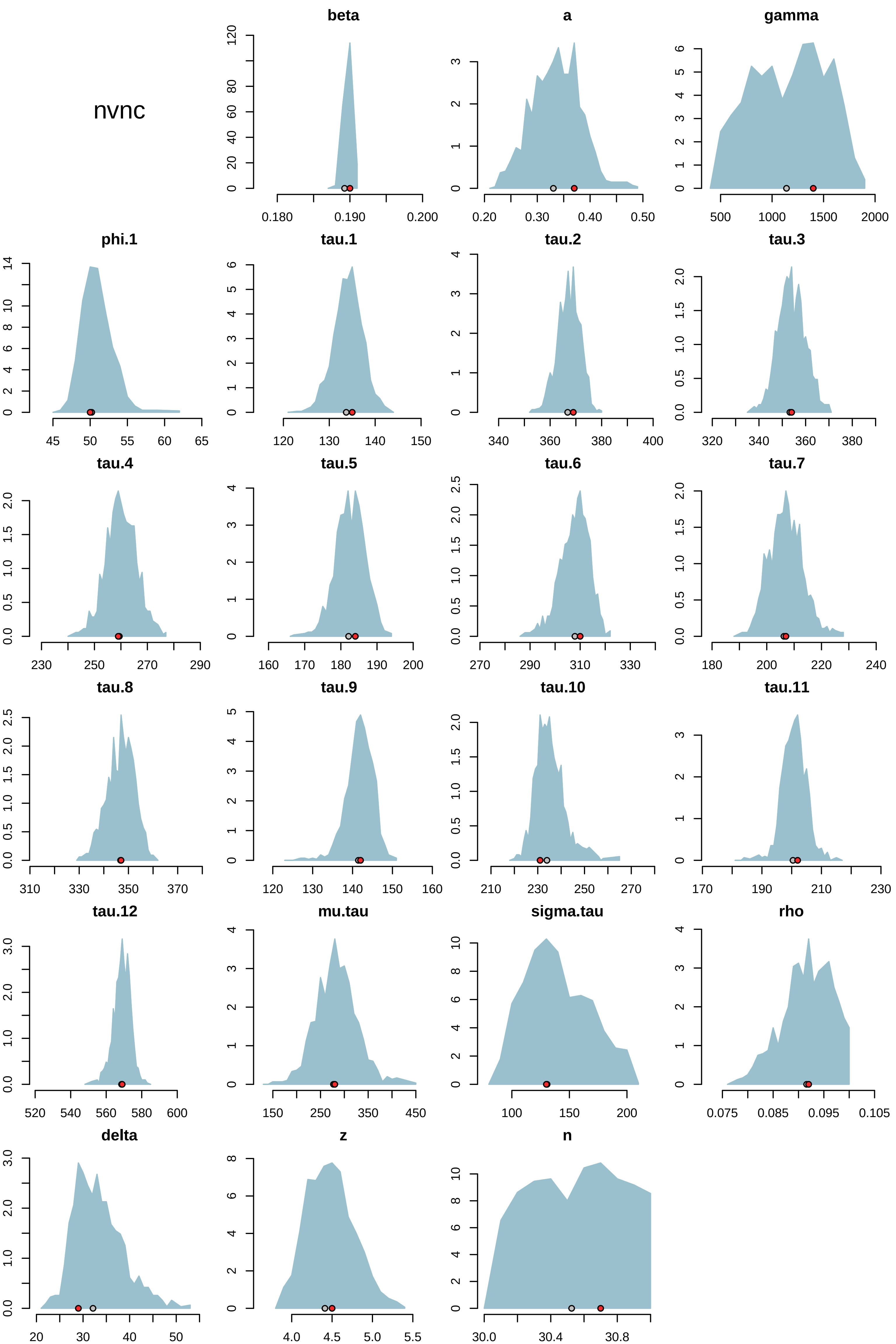
nvnc
beta
0.180 0.190 0.200
a
0.20 0.30 0.40 0.50
gamma
500 1000 1500 2000
phi.1
45 50 55 60 65
tau.1
120 130 140 150
tau.2
340 360 380 400
tau.3
320 340 360 380
tau.4
230 250 270 290
tau.5
160 170 180 190 200
tau.6
270 290 310 330
tau.7
180 200 220 240
tau.8
310 330 350 370
tau.9
120 130 140 150 160
tau.10
210 230 250 270
tau.11
170 190 210 230
tau.12
520 540 560 580 600
mu.tau
150 250 350 450
sigma.tau
100 150 200
rho
0.075 0.085 0.095 0.105
delta
20 30 40 50
z
4.0 4.5 5.0 5.5
n
30.0 30.4 30.8

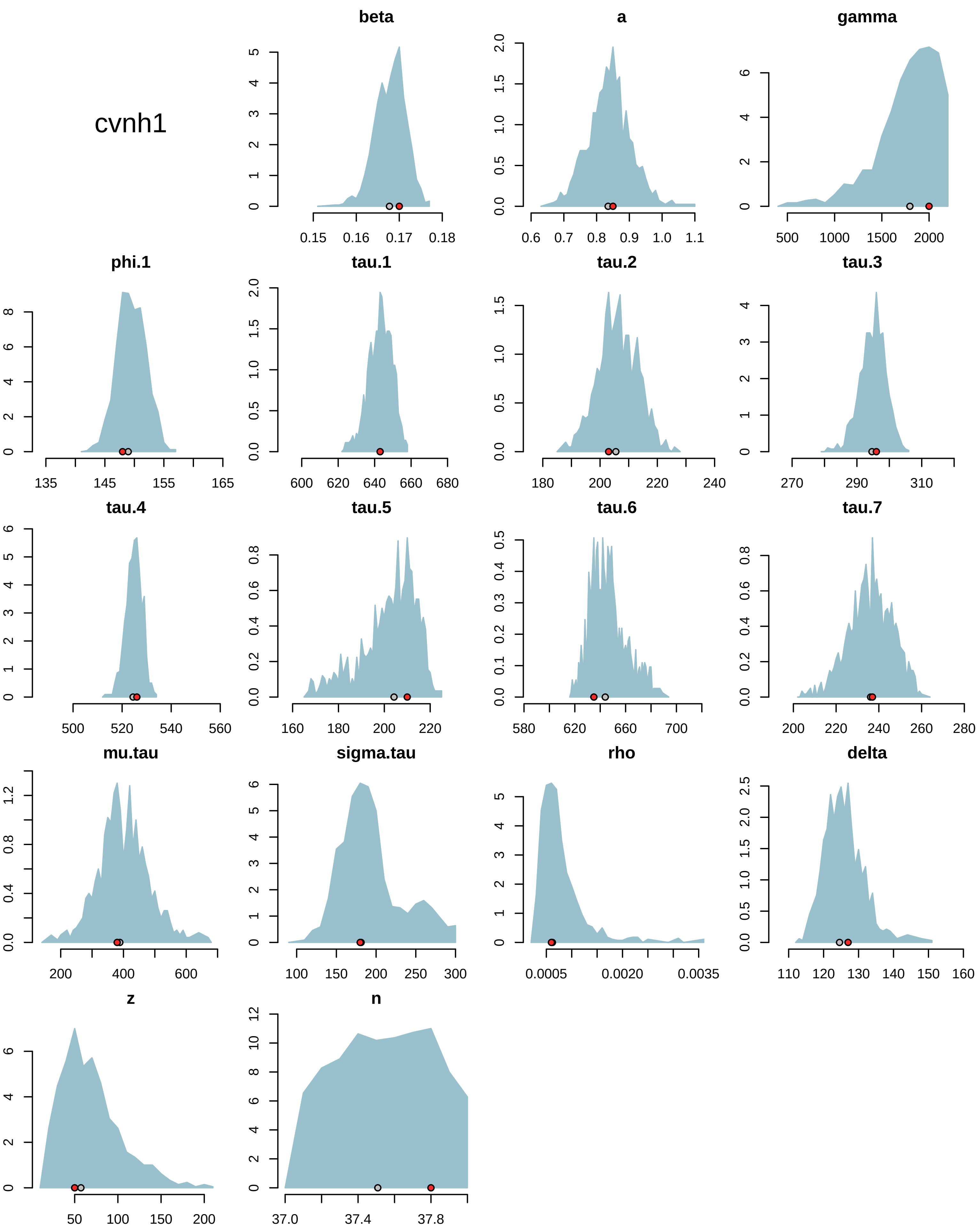
cvnh1
beta
0.15 0.16 0.17 0.18
a
0.6 0.7 0.8 0.9 1.0 1.1
gamma
500 1000 1500 2000
phi.1
135 145 155 165
tau.1
600 620 640 660 680
tau.2
180 200 220 240
tau.3
270 290 310
tau.4
500 520 540 560
tau.5
160 180 200 220
tau.6
580 620 660 700
tau.7
200 220 240 260 280
mu.tau
200 400 600
sigma.tau
100 150 200 250 300
rho
0.0005 0.0020 0.0035
delta
110 120 130 140 150 160
z
50 100 150 200
n
37.0 37.4 37.8

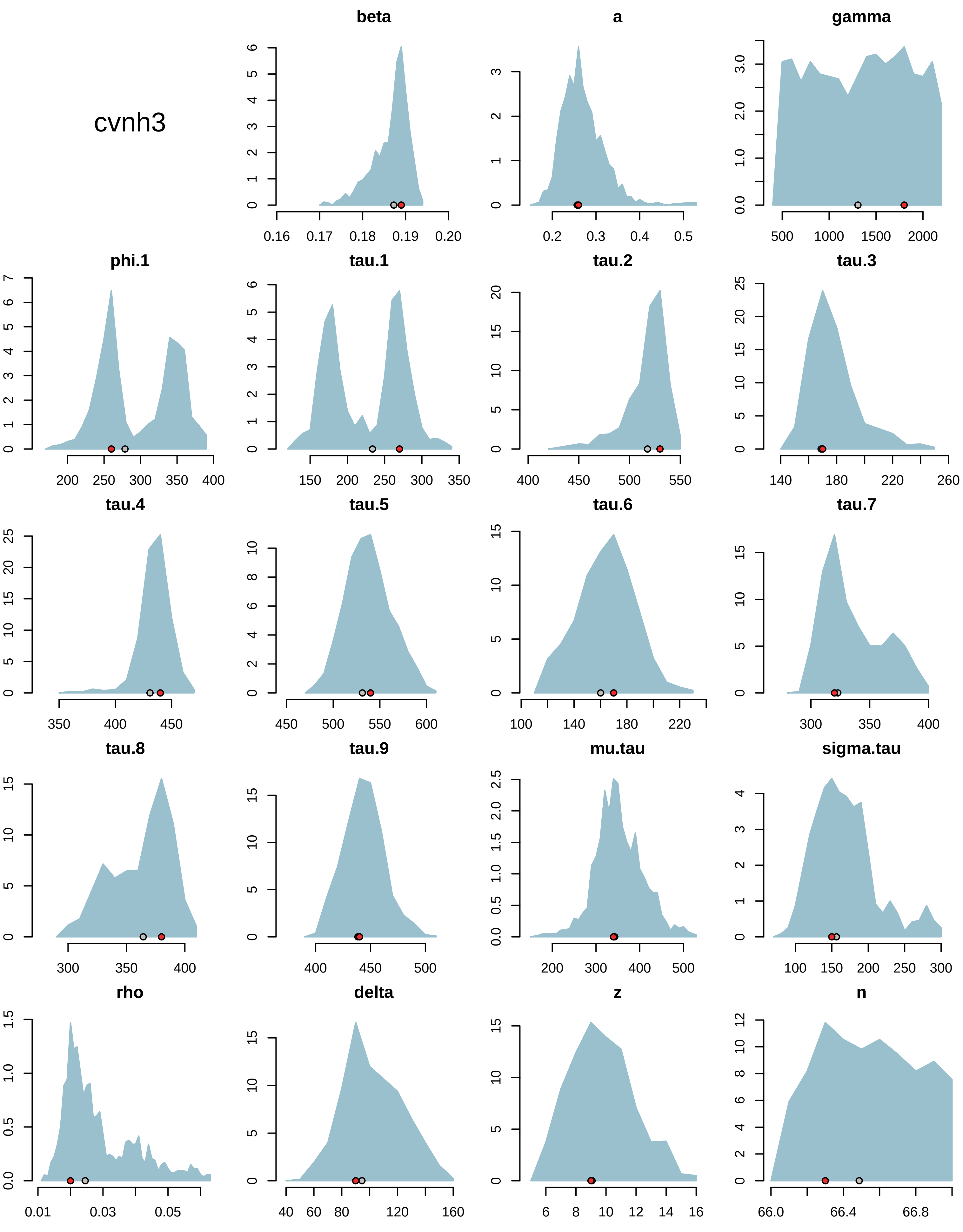
cvnh3
beta
a
gamma
phi.1
tau.1
tau.2
tau.3
tau.4
tau.5
tau.6
tau.7
tau.8
tau.9
mu.tau
sigma.tau
rho
delta
z
n

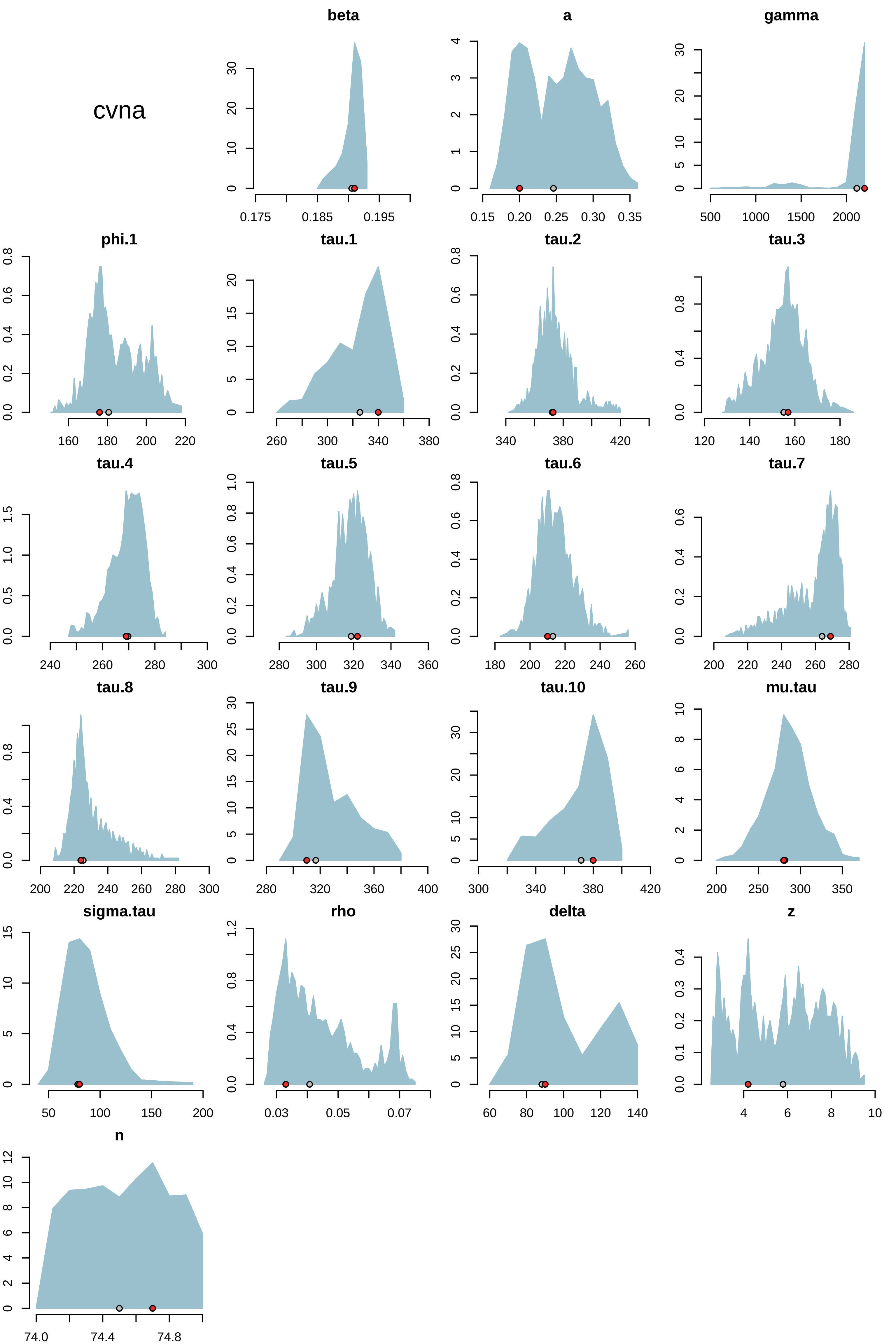
cvna
beta
a
gamma
phi.1
tau.1
tau.2
tau.3
tau.4
tau.5
tau.6
tau.7
tau.8
tau.9
tau.10
mu.tau
sigma.tau
rho
delta
z
n

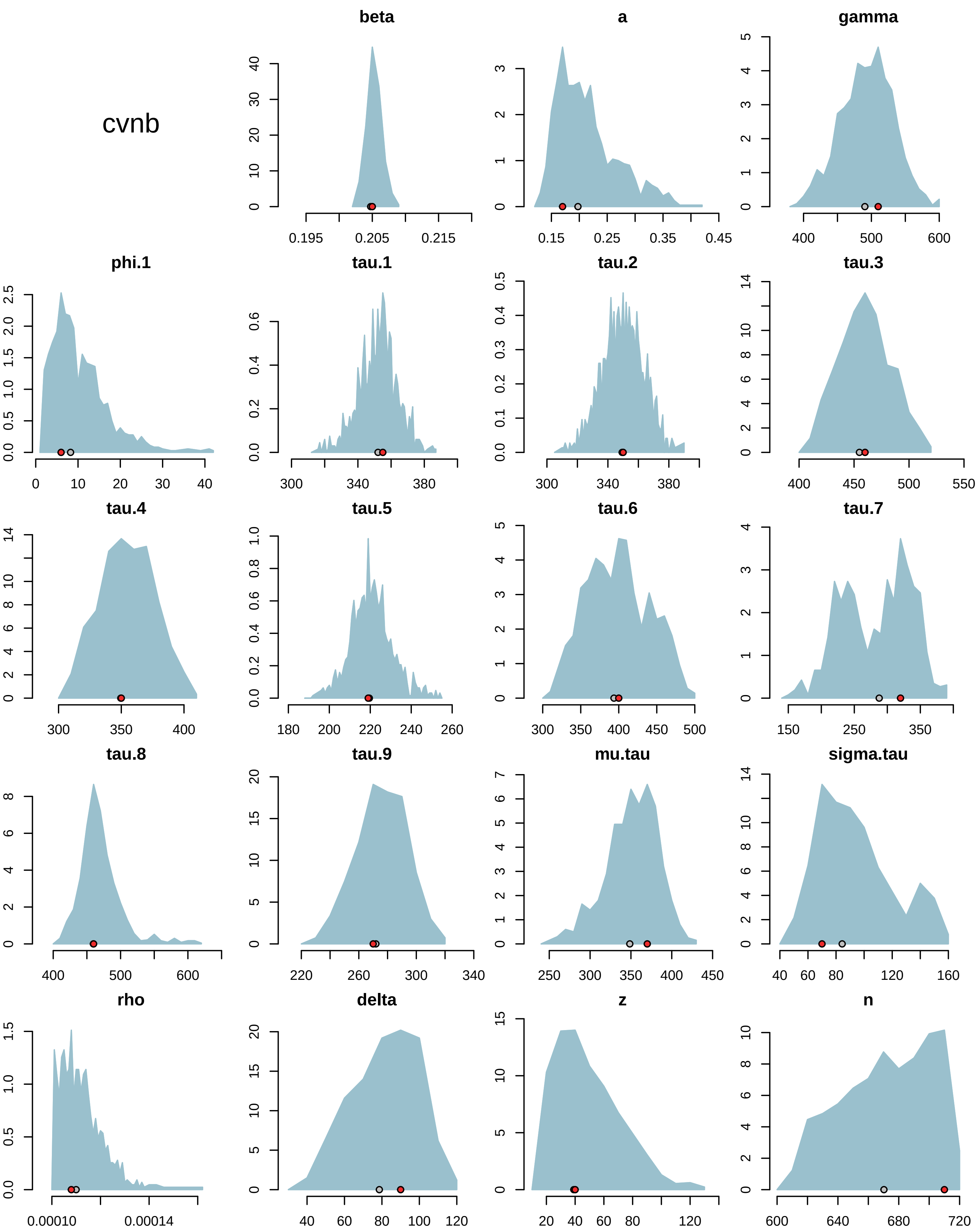
cvnb
beta
a
gamma
phi.1
tau.1
tau.2
tau.3
tau.4
tau.5
tau.6
tau.7
tau.8
tau.9
mu.tau
sigma.tau
rho
delta
z
n

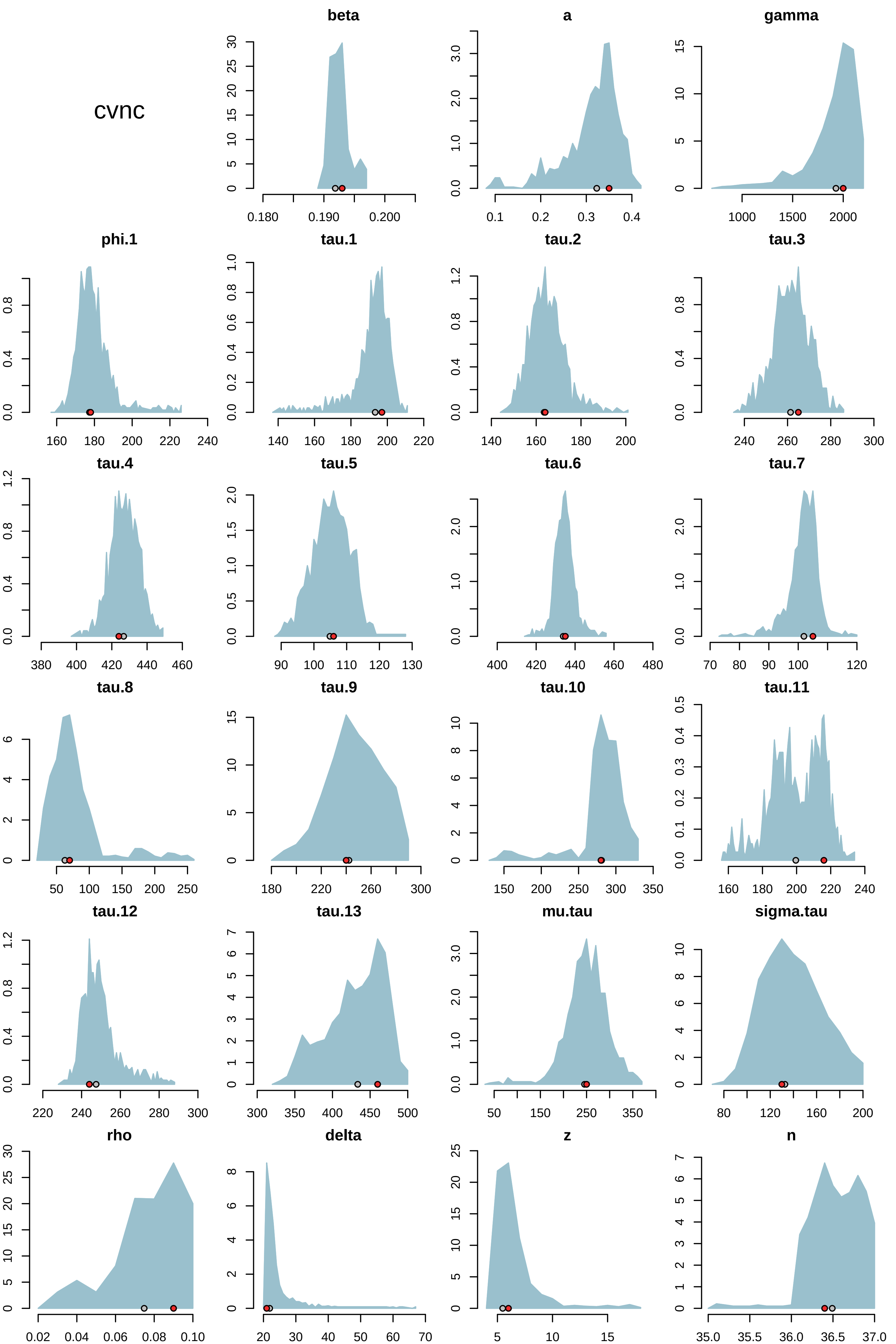
cvnc
beta
0 5 10 15 20 25 30
0.180 0.190 0.200
a
0.0 1.0 2.0 3.0
0.1 0.2 0.3 0.4
gamma
0 5 10 15
1000 1500 2000
phi.1
0.0 0.4 0.8
160 180 200 220 240
tau.1
0.0 0.2 0.4 0.6 0.8 1.0
140 160 180 200 220
tau.2
0.0 0.4 0.8 1.2
140 160 180 200
tau.3
0.0 0.4 0.8
240 260 280 300
tau.4
0.0 0.4 0.8 1.2
380 400 420 440 460
tau.5
0.0 0.5 1.0 1.5 2.0
90 100 110 120 130
tau.6
0.0 1.0 2.0
400 420 440 460 480
tau.7
0.0 1.0 2.0
70 80 90 100 120
tau.8
0 2 4 6
50 100 150 200 250
tau.9
0 5 10 15
180 220 260 300
tau.10
0 2 4 6 8 10
150 200 250 300 350
tau.11
0.0 0.1 0.2 0.3 0.4 0.5
160 180 200 220 240
tau.12
0.0 0.4 0.8 1.2
220 240 260 280 300
tau.13
0 1 2 3 4 5 6 7
300 350 400 450 500
mu.tau
0.0 1.0 2.0 3.0
50 150 250 350
sigma.tau
0 2 4 6 8 10
80 120 160 200
rho
0 5 10 15 20 25 30
0.02 0.04 0.06 0.08 0.10
delta
0 2 4 6 8
20 30 40 50 60 70
z
0 5 10 15 20 25
5 10 15
n
0 1 2 3 4 5 6 7
35.0 35.5 36.0 36.5 37.0

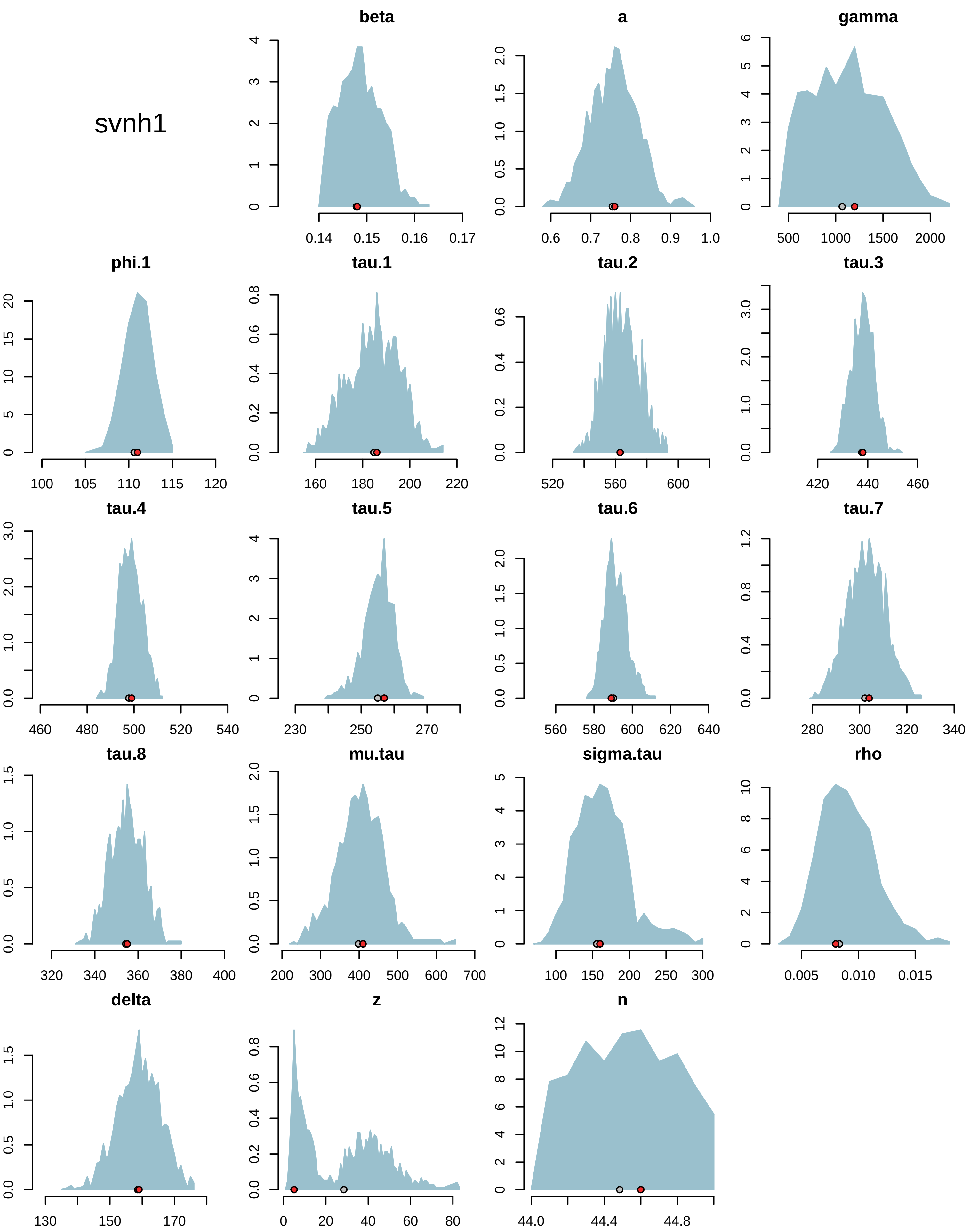

svnh1
beta
a
gamma
phi.1
tau.1
tau.2
tau.3
tau.4
tau.5
tau.6
tau.7
tau.8
mu.tau
sigma.tau
rho
delta
z
n

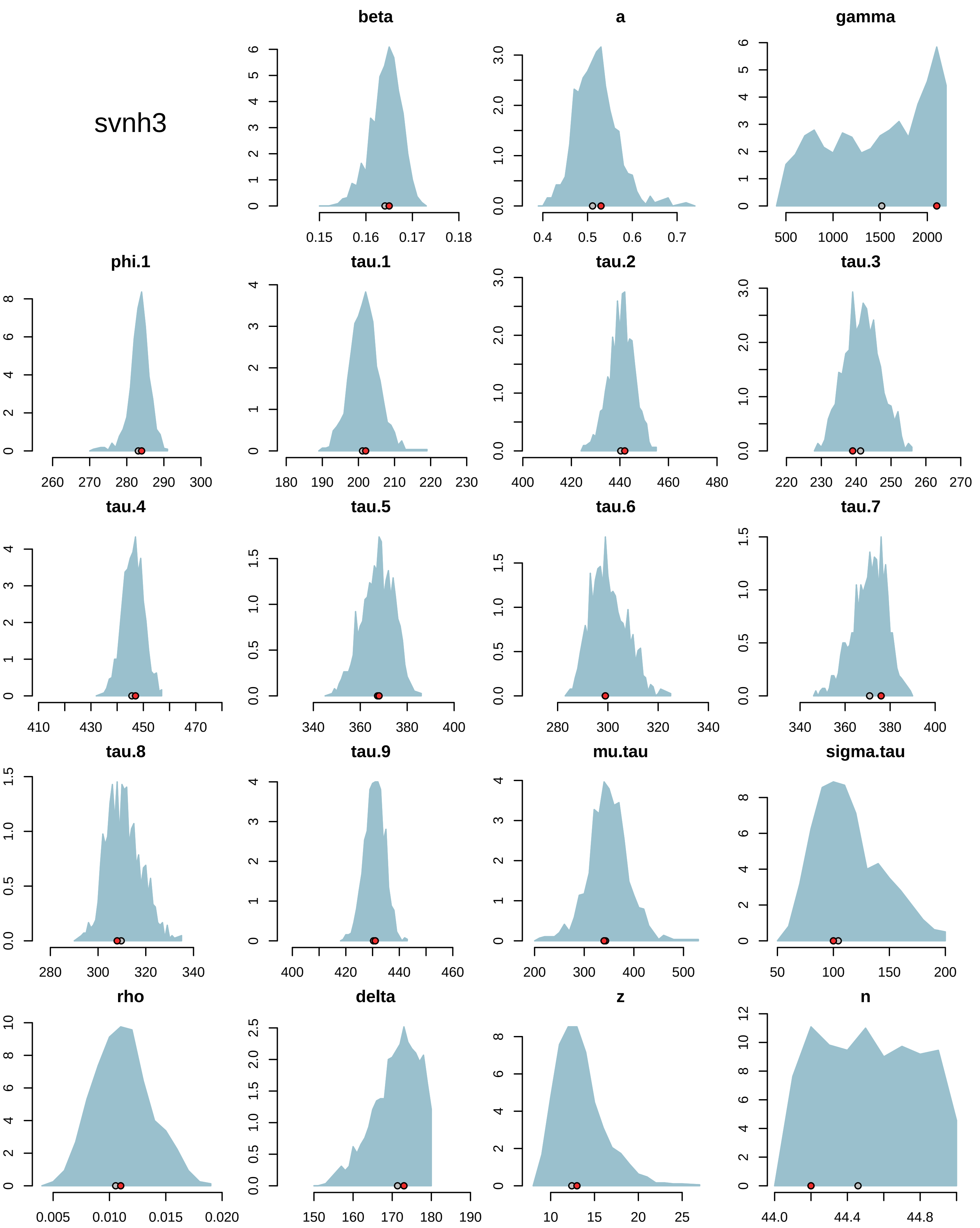

svnh3
beta
a
gamma
phi.1
tau.1
tau.2
tau.3
tau.4
tau.5
tau.6
tau.7
tau.8
tau.9
mu.tau
sigma.tau
rho
delta
z
n

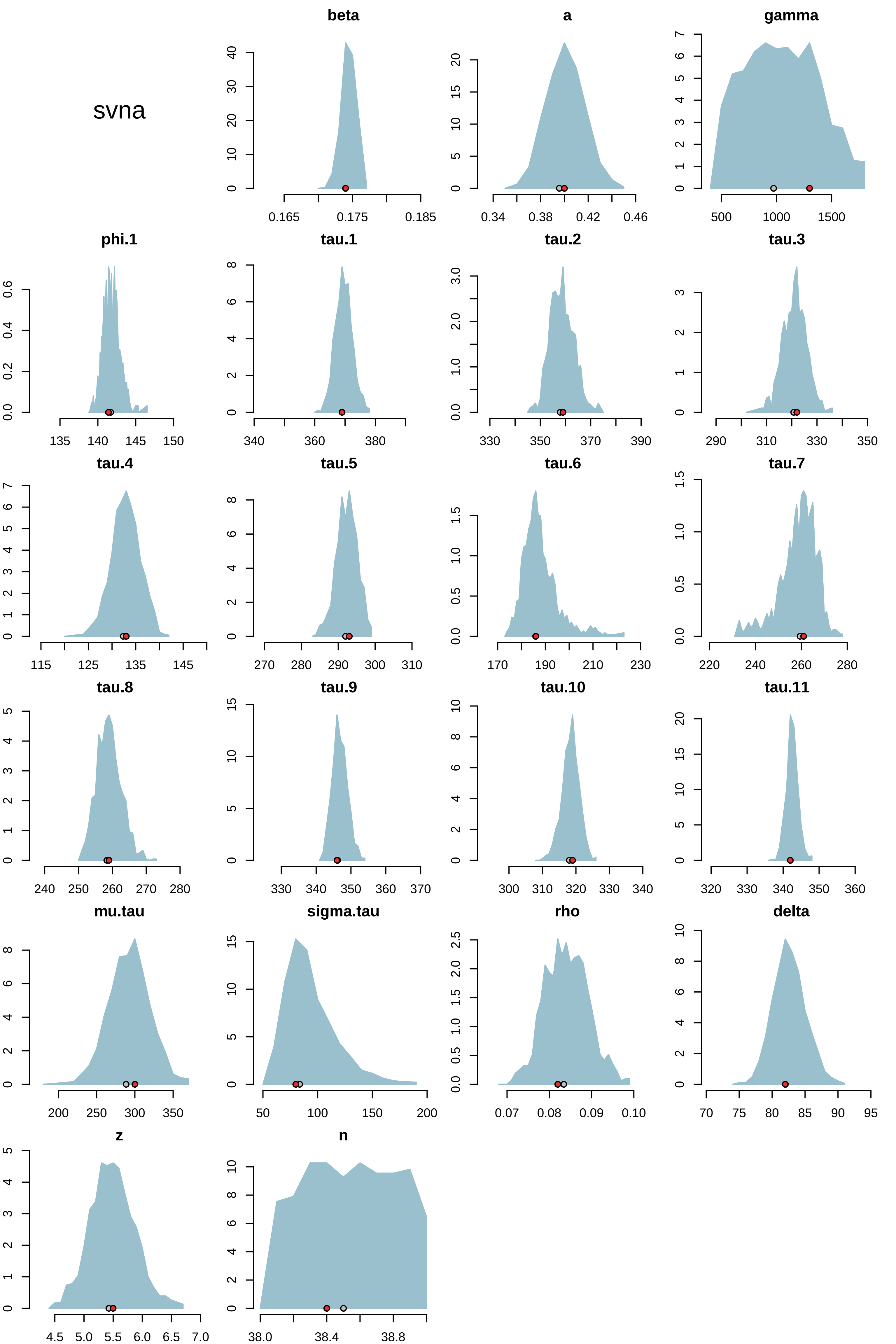
svna
beta
a
gamma
phi.1
tau.1
tau.2
tau.3
tau.4
tau.5
tau.6
tau.7
tau.8
tau.9
tau.10
tau.11
mu.tau
sigma.tau
rho
delta
z
n

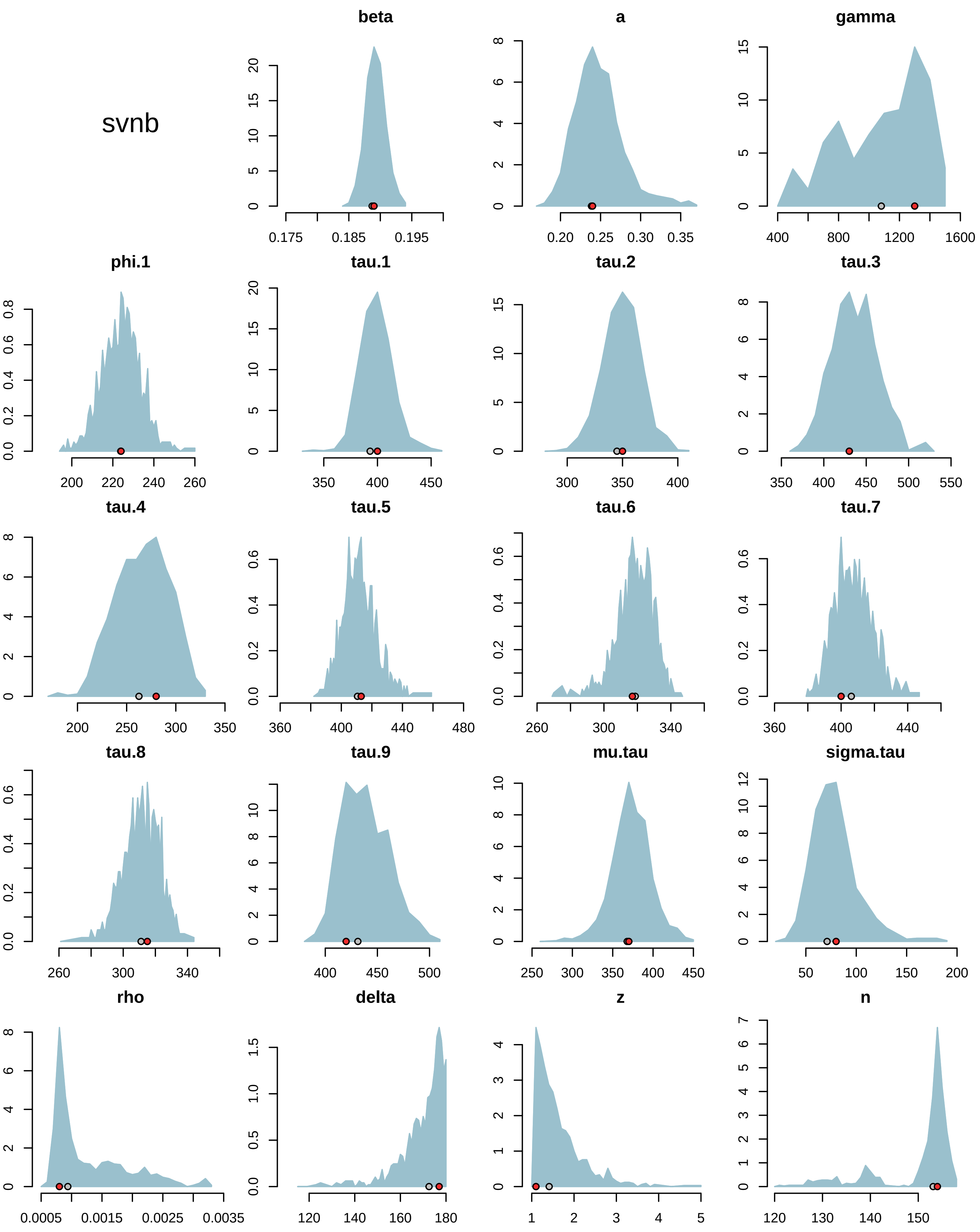
svnb
beta
0.175 0.185 0.195
a
0.20 0.25 0.30 0.35
gamma
400 800 1200 1600
phi.1
200 220 240 260
tau.1
350 400 450
tau.2
300 350 400
tau.3
350 400 450 500 550
tau.4
200 250 300 350
tau.5
360 400 440 480
tau.6
260 300 340
tau.7
360 400 440
tau.8
260 300 340
tau.9
400 450 500
mu.tau
250 300 350 400 450
sigma.tau
50 100 150 200
rho
0.0005 0.0015 0.0025 0.0035
delta
120 140 160 180
z
1 2 3 4 5
n
120 130 140 150

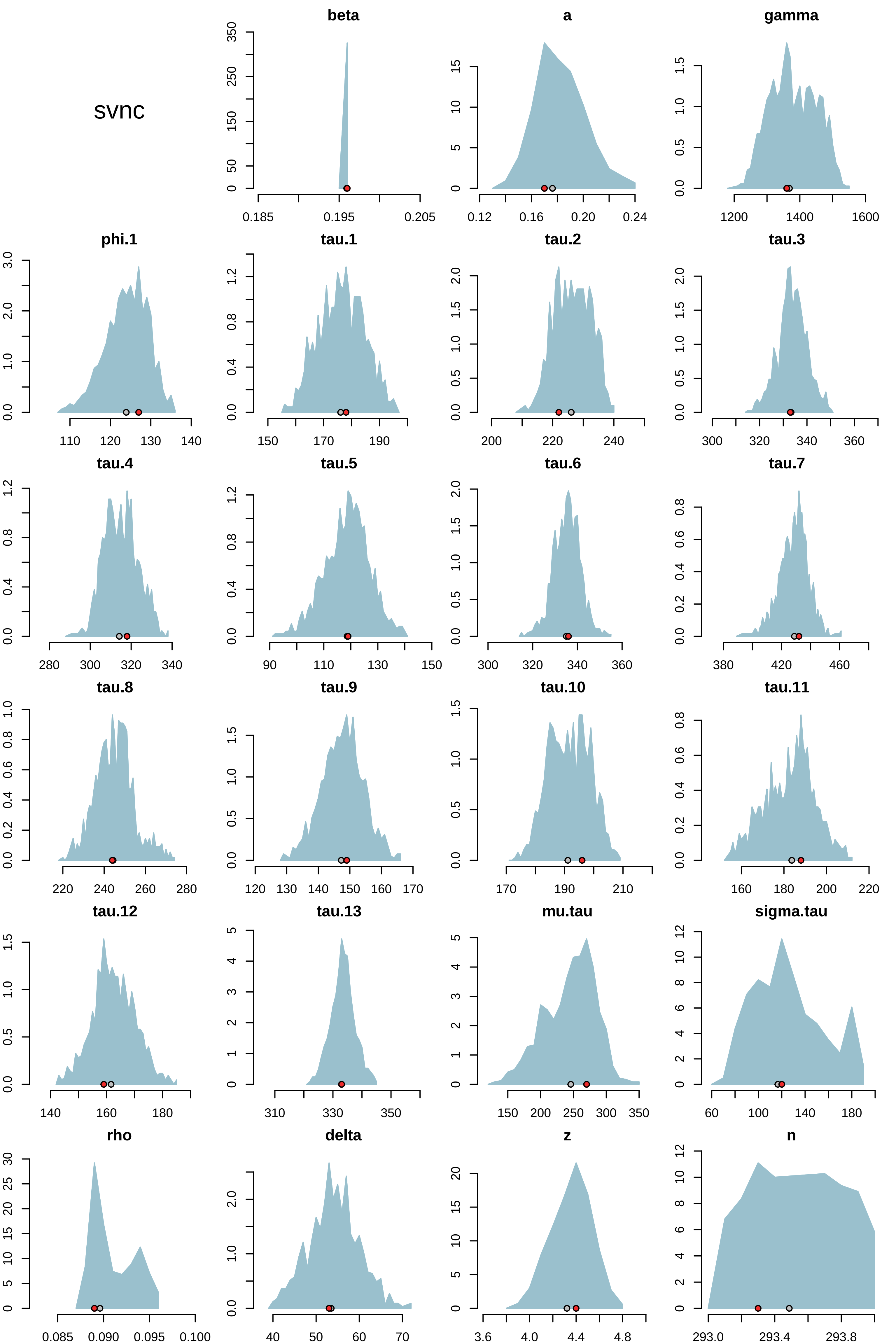

svnc
beta
0.185 0.195 0.205
a
0.12 0.16 0.20 0.24
gamma
1200 1400 1600
phi.1
110 120 130 140
tau.1
150 170 190
tau.2
200 220 240
tau.3
300 320 340 360
tau.4
280 300 320 340
tau.5
90 110 130 150
tau.6
300 320 340 360
tau.7
380 420 460
tau.8
220 240 260 280
tau.9
120 130 140 150 160 170
tau.10
170 190 210
tau.11
160 180 200 220
tau.12
140 160 180
tau.13
310 330 350
mu.tau
150 200 250 300 350
sigma.tau
60 100 140 180
rho
0.085 0.090 0.095 0.100
delta
40 50 60 70
z
3.6 4.0 4.4 4.8
n
293.0 293.4 293.8

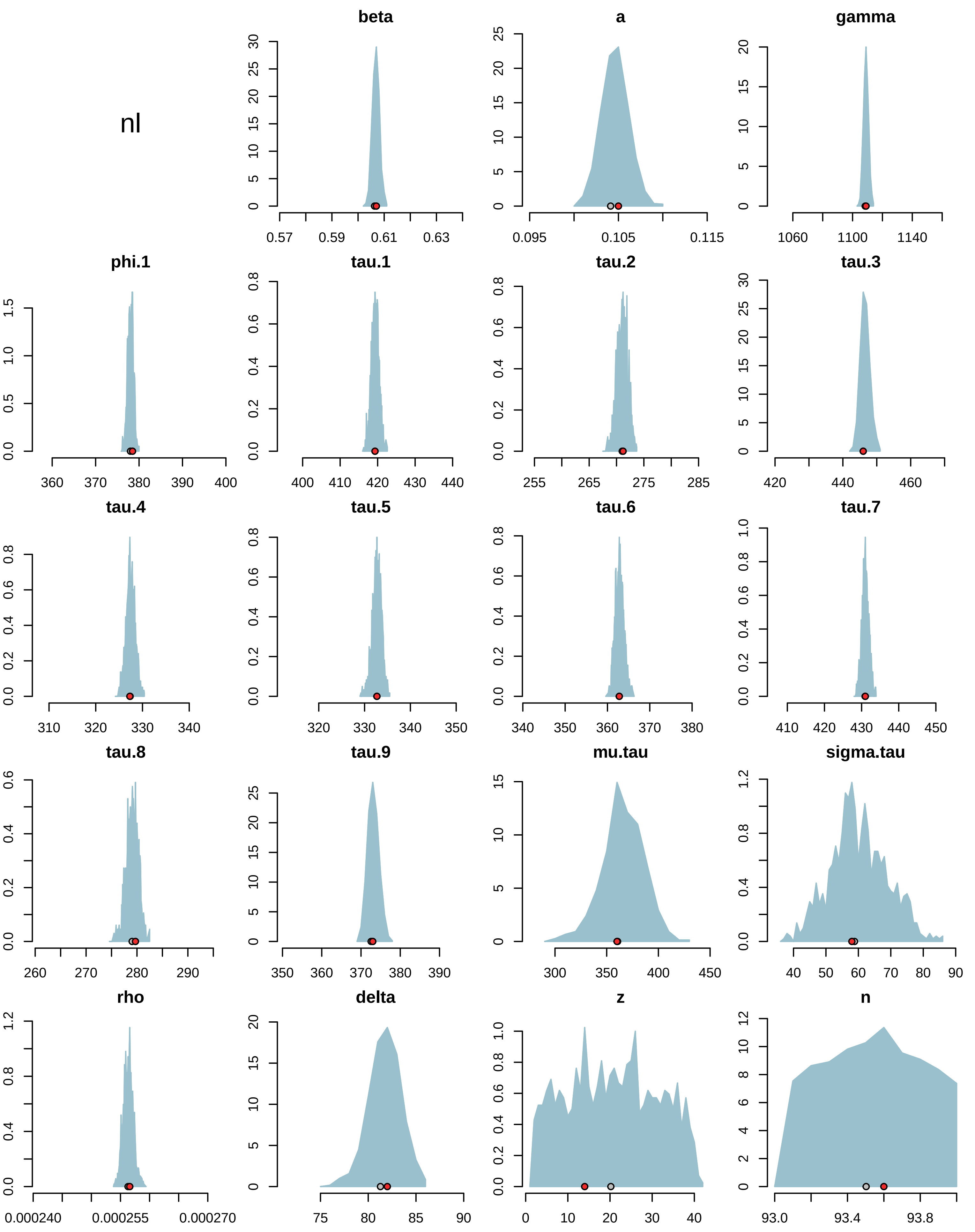

nl
beta
a
gamma
phi.1
tau.1
tau.2
tau.3
tau.4
tau.5
tau.6
tau.7
tau.8
tau.9
mu.tau
sigma.tau
rho
delta
z
n

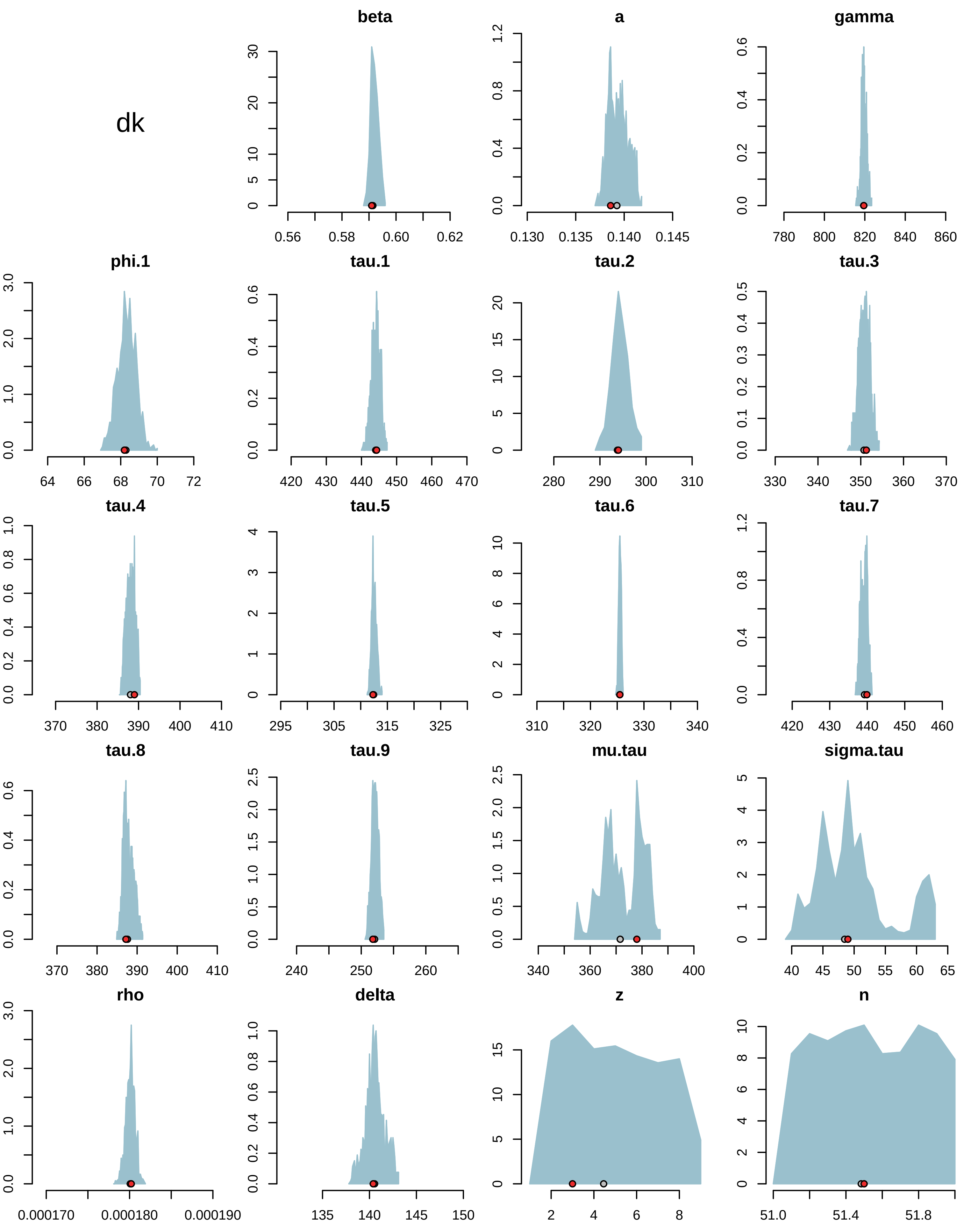
dk
beta
a
gamma
phi.1
tau.1
tau.2
tau.3
tau.4
tau.5
tau.6
tau.7
tau.8
tau.9
mu.tau
sigma.tau
rho
delta
z
n

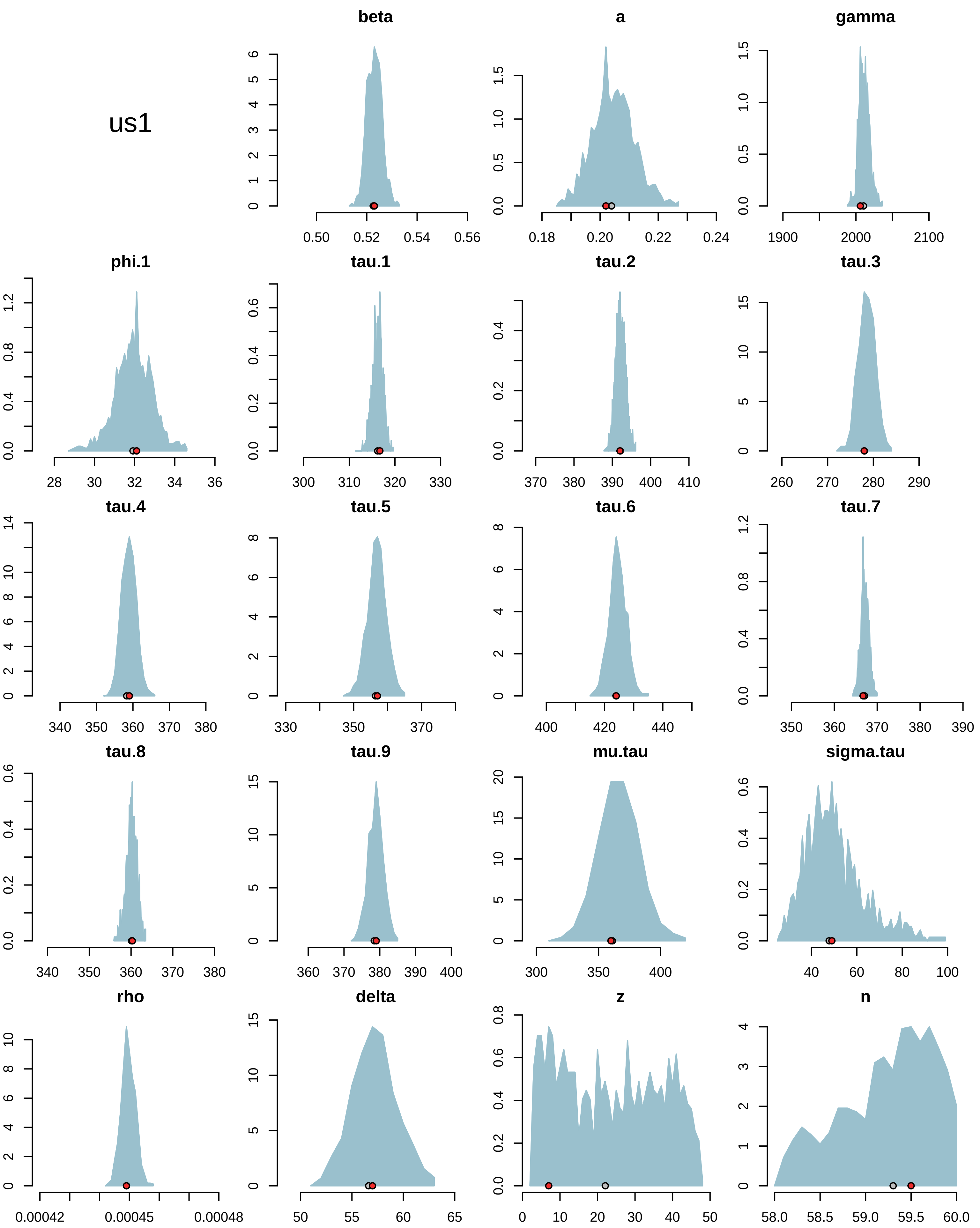
us1
beta
a
gamma
phi.1
tau.1
tau.2
tau.3
tau.4
tau.5
tau.6
tau.7
tau.8
tau.9
mu.tau
sigma.tau
rho
delta
z
n

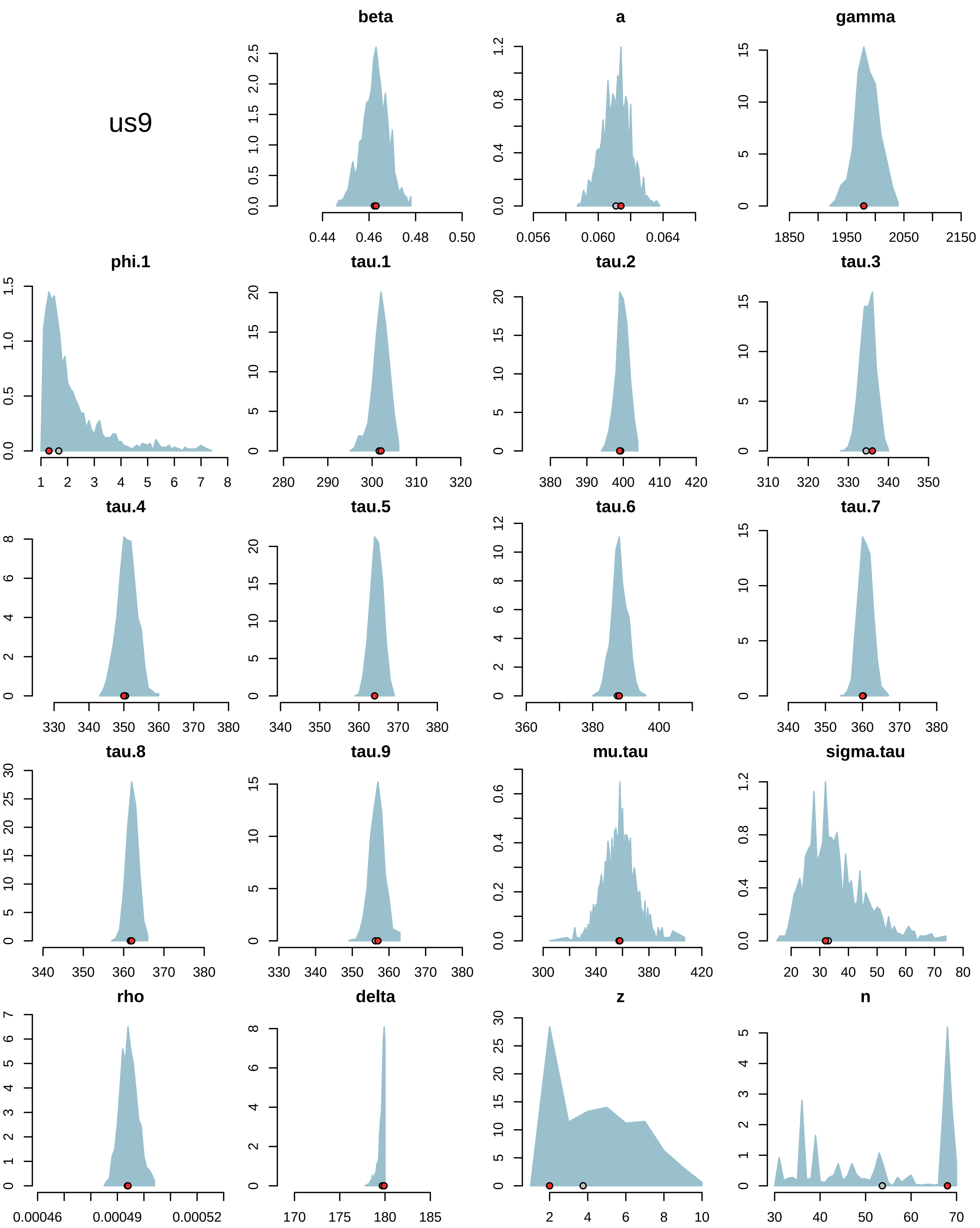
us9
beta
a
gamma
phi.1
tau.1
tau.2
tau.3
tau.4
tau.5
tau.6
tau.7
tau.8
tau.9
mu.tau
sigma.tau
rho
delta
z
n
