# Supplementary material for: Repeatability and timing of tropical influenza epidemics
Source: PLoS Comput Biol. 2023 Jul 19;19(7):e1011317. doi: 10.1371/journal.pcbi.1011317 (PMC10389745; doi:10.1371/journal.pcbi.1011317)
Supplement: S1 Table — The row for tau_k indicates that all τ parameters were given the same prior distribution and standard deviation for the proposal distribution. (PDF) [file pcbi.1011317.s006.pdf]

Parameter	Prior distribution	Proposal distribution standard deviation
beta	U(0.14, 1)	0.01
amp	Exp(1)	0.01
1/gamma	U(180, 365x6)	10
phi.1	U(0, 450)	5
tau_k	U(20, 700)	5
mu.tau	U(20, 700)	5
sigma.tau	U(1, 200)	2
rho (reporting)	U(0.0001, 0.1)	0.0002
delta (epidemic duration)	U(20, 180)	2
z (epidemic end)	U(0, 1000)	5
n (importation)	U(30, 1000)	5
