# Supplementary material for: Repeatability and timing of tropical influenza epidemics
Source: PLoS Comput Biol. 2023 Jul 19;19(7):e1011317. doi: 10.1371/journal.pcbi.1011317 (PMC10389745; doi:10.1371/journal.pcbi.1011317)
Supplement: S2 Table — Standardized mean absolute error (sMAE) values indicate relative goodness of fit across models. (PDF) [file pcbi.1011317.s007.pdf]

param	Estimate_nvn_h1	CrI_nvn_h1	Estimate_nvn_h3	CrI_nvn_h3	Estimate_nvn_a	CrI_nvn_a
beta	0.1902	[0.1826, 0.1989]	0.1927	[0.1868, 0.2003]	0.1855	[0.1821, 0.1871]
a	0.8672	[0.7676, 0.9544]	1.1022	[1.0135, 1.1591]	0.3204	[0.2724, 0.3799]
$gamma^{-1}$	1039	[625, 2103]	1160	[940, 1963]	1382	[438, 1938]
phi.1	118	[112, 125]	351	[344, 356]	132	[126, 141]
tau.1	232	[196, 249]	184	[177, 191]	409	[399, 416]
tau.2	542	[523, 579]	302	[285, 325]	367	[360, 371]
tau.3	424	[415, 433]	330	[305, 344]	257	[248, 272]
tau.4	481	[471, 497]	471	[464, 479]	161	[148, 170]
tau.5	231	[195, 249]	654	[650, 660]	316	[308, 325]
tau.6	527	[511, 559]	562	[532, 569]	174	[162, 183]
tau.7	364	[356, 371]	156	[149, 188]	477	[469, 489]
tau.8	413	[400, 423]	370	[366, 374]	276	[265, 285]
tau.9	NA	NA	NA	NA	304	[295, 313]
tau.10	NA	NA	NA	NA	NA	NA
tau.11	NA	NA	NA	NA	NA	NA
tau.12	NA	NA	NA	NA	NA	NA
tau.13	NA	NA	NA	NA	NA	NA
mu.tau	386	[282, 506]	376	[249, 503]	302	[228, 398]
sigma.tau	124	[88, 260]	173	[118, 270]	106	[73, 181]
rho	0.00015	[0.00011, 0.00036]	0.0001	[0.0001, 0.00018]	0.06205	[0.05007, 0.07652]
delta	89.4	[79.6, 97.9]	81.7	[75.9, 86.4]	73.8	[62.7, 86.6]
z	165	[87, 526]	509	[332, 673]	3	[2, 3]
n	31.79	[31.05, 31.96]	42.66	[42.04, 42.96]	31.52	[31.04, 31.95]
sMAE	0.0412		0.0454		0.0741	

param	Estimate_nvn_b	CrI_nvn_b	Estimate_nvn_ili+	CrI_nvn_ili+
beta	0.1987	[0.1966, 0.2004]	0.1893	[0.1882, 0.1903]
a	0.4657	[0.3205, 0.9147]	0.3329	[0.2415, 0.4114]
$gamma^{-1}$	396	[369, 490]	1299	[429, 1721]
phi.1	38	[31, 45]	50	[47, 54]
tau.1	387	[329, 491]	134	[127, 140]
tau.2	250	[136, 307]	367	[358, 375]
tau.3	487	[451, 524]	352	[342, 365]
tau.4	352	[333, 374]	259	[248, 271]
tau.5	430	[399, 459]	184	[174, 190]
tau.6	242	[218, 275]	309	[295, 317]
tau.7	376	[340, 440]	206	[196, 219]
tau.8	216	[150, 252]	348	[335, 356]
tau.9	654	[562, 690]	141	[134, 147]
tau.10	NA	NA	233	[225, 248]
tau.11	NA	NA	201	[193, 208]
tau.12	NA	NA	568	[558, 577]
tau.13	NA	NA	NA	NA
mu.tau	374	[272, 470]	276	[196, 365]
sigma.tau	148	[92, 193]	126	[90, 192]
rho	0.0001	[0.0001, 0.00012]	0.0908	[0.08105, 0.0993]
delta	52.6	[29, 91.6]	29.5	[24.9, 44.3]
z	3	[1, 17]	4	[4, 5]
n	592.51	[180.56, 958.29]	30.67	[30.04, 30.96]
sMAE	0.0593		0.1034	

param	Estimate_cvn_h1	CrI_cvn_h1	Estimate_cvn_h3	CrI_cvn_h3	Estimate_cvn_a	CrI_cvn_a
beta	0.1691	[0.16, 0.1738]	0.1882	[0.1757, 0.1919]	0.1909	[0.186, 0.1923]
a	0.8376	[0.7119, 0.9649]	0.2513	[0.1923, 0.362]	0.1979	[0.1743, 0.3287]
$gamma^{-1}$	1904	[964, 2168]	1622	[435, 2128]	2154	[1133, 2187]
phi.1	148	[144, 153]	253	[211, 372]	175	[162, 208]
tau.1	643	[629, 654]	261	[146, 301]	333	[272, 349]
tau.2	205	[192, 219]	521	[458, 540]	371	[354, 409]
tau.3	295	[286, 302]	163	[147, 219]	156	[134, 172]
tau.4	525	[518, 531]	431	[400, 454]	271	[254, 279]
tau.5	209	[173, 218]	527	[489, 582]	320	[297, 334]
tau.6	646	[624, 678]	163	[118, 199]	210	[197, 236]
tau.7	235	[215, 255]	311	[296, 385]	269	[222, 276]
tau.8	NA	NA	375	[304, 395]	223	[214, 259]
tau.9	NA	NA	439	[406, 478]	308	[298, 368]
tau.10	NA	NA	NA	NA	378	[325, 390]
tau.11	NA	NA	NA	NA	NA	NA
tau.12	NA	NA	NA	NA	NA	NA
tau.13	NA	NA	NA	NA	NA	NA
mu.tau	369	[238, 554]	335	[244, 468]	277	[229, 335]
sigma.tau	179	[129, 281]	143	[99, 277]	70	[51, 126]
rho	0.0005	[0.00028, 0.00191]	0.0203	[0.015, 0.0567]	0.0325	[0.02817, 0.06924]
delta	124.5	[115.9, 137.2]	85.7	[59.1, 139.4]	81	[66.7, 134.1]
z	46	[16, 145]	9	[6, 14]	4	[3, 9]
n	37.61	[37.04, 37.96]	66.26	[66.04, 66.96]	74.67	[74.05, 74.95]
sMAE	0.0265		0.0636		0.0562	

param	Estimate_cvn_b	CrI_cvn_b	Estimate_cvn_ili+	CrI_cvn_ili+
beta	0.2047	[0.2026, 0.2073]	0.1923	[0.1899, 0.1964]
a	0.165	[0.1355, 0.3388]	0.3411	[0.1665, 0.3888]
$gamma^{-1}$	500	[411, 560]	1945	[1143, 2152]
phi.1	6	[2, 25]	176	[166, 203]
tau.1	354	[328, 373]	194	[160, 204]
tau.2	350	[323, 374]	162	[151, 183]
tau.3	457	[411, 502]	262	[244, 277]
tau.4	346	[309, 391]	425	[411, 442]
tau.5	219	[201, 243]	105	[93, 115]
tau.6	398	[320, 473]	434	[425, 445]
tau.7	319	[184, 360]	102	[88, 108]
tau.8	455	[418, 553]	64	[26, 216]
tau.9	278	[237, 304]	237	[197, 281]
tau.10	NA	NA	271	[154, 321]
tau.11	NA	NA	214	[164, 223]
tau.12	NA	NA	247	[236, 276]
tau.13	NA	NA	458	[348, 482]
mu.tau	363	[274, 400]	241	[158, 331]
sigma.tau	68	[50, 147]	124	[93, 189]
rho	0.00011	[0.0001, 0.00013]	0.08763	[0.02189, 0.09734]
delta	89	[43.6, 107.2]	20.8	[20.1, 41.6]
z	30	[11, 95]	5	[4, 14]
n	699.26	[612.2, 710.52]	36.37	[36.01, 36.96]
sMAE	0.1041		0.0757	

param	Estimate_svn_h1	CrI_svn_h1	Estimate_svn_h3	CrI_svn_h3	Estimate_svn_a	CrI_svn_a
beta	0.1476	[0.1409, 0.1569]	0.1649	[0.1564, 0.1693]	0.1738	[0.1719, 0.1758]
a	0.7592	[0.6426, 0.8598]	0.5154	[0.4305, 0.6068]	0.3967	[0.3652, 0.4251]
$gamma^{-1}$	1110	[425, 1797]	2034	[428, 2170]	886	[413, 1662]
phi.1	111	[108, 114]	283	[277, 288]	142	[140, 144]
tau.1	185	[163, 203]	201	[193, 209]	369	[364, 375]
tau.2	560	[545, 585]	441	[429, 450]	358	[350, 368]
tau.3	438	[429, 446]	242	[232, 251]	321	[311, 329]
tau.4	497	[489, 508]	446	[438, 453]	132	[126, 138]
tau.5	256	[244, 262]	367	[353, 378]	292	[286, 297]
tau.6	589	[581, 603]	298	[288, 314]	185	[177, 208]
tau.7	302	[287, 317]	373	[355, 382]	261	[238, 270]
tau.8	354	[340, 369]	309	[298, 325]	258	[252, 266]
tau.9	NA	NA	431	[423, 437]	346	[343, 351]
tau.10	NA	NA	NA	NA	318	[312, 323]
tau.11	NA	NA	NA	NA	342	[339, 346]
tau.12	NA	NA	NA	NA	NA	NA
tau.13	NA	NA	NA	NA	NA	NA
mu.tau	394	[276, 515]	335	[259, 418]	291	[235, 340]
sigma.tau	142	[97, 252]	98	[65, 176]	76	[56, 144]
rho	0.00771	[0.00439, 0.01425]	0.01044	[0.00621, 0.01581]	0.08228	[0.07389, 0.09414]
delta	158.5	[145.4, 171.1]	172.2	[157.2, 179.4]	81.6	[77.7, 87.1]
z	6	[3, 64]	12	[9, 19]	5	[5, 6]
n	44.55	[44.04, 44.95]	44.41	[44.04, 44.94]	38.57	[38.03, 38.96]
sMAE	0.0417		0.0461		0.0689	

param	Estimate_svn_b	CrI_svn_b	Estimate_svn_ili+	CrI_svn_ili+
beta	0.1887	[0.1857, 0.192]	0.1959	[0.1956, 0.1963]
a	0.2345	[0.1936, 0.3102]	0.1658	[0.1442, 0.2211]
$gamma^{-1}$	1258	[383, 1443]	1355	[1253, 1499]
phi.1	225	[205, 241]	125	[113, 132]
tau.1	392	[367, 426]	176	[161, 191]
tau.2	348	[310, 382]	227	[216, 236]
tau.3	424	[381, 483]	332	[321, 346]
tau.4	269	[210, 308]	310	[301, 331]
tau.5	410	[392, 435]	120	[101, 133]
tau.6	318	[294, 336]	335	[323, 346]
tau.7	403	[387, 430]	431	[408, 446]
tau.8	311	[288, 331]	248	[228, 265]
tau.9	429	[398, 481]	148	[134, 160]
tau.10	NA	NA	195	[178, 204]
tau.11	NA	NA	187	[159, 204]
tau.12	NA	NA	159	[148, 177]
tau.13	NA	NA	333	[325, 341]
mu.tau	366	[319, 418]	261	[156, 303]
sigma.tau	64	[39, 127]	115	[74, 180]
rho	0.00076	[0.00064, 0.00272]	0.08833	[0.0878, 0.09505]
delta	176.5	[147.3, 179.6]	53.3	[42.4, 64.6]
z	1	[1, 3]	4	[4, 5]
n	153.46	[127.85, 156.75]	293.28	[293.05, 293.96]
sMAE	0.0851		0.0693	

param	Estimate_nl_ili+	CrI_nl_ili+	Estimate_dk_ili+	CrI_dk_ili+
beta	0.6063	[0.6038, 0.6091]	0.5906	[0.589, 0.5944]
a	0.1042	[0.1013, 0.1071]	0.1385	[0.1377, 0.1412]
$gamma^{-1}$	1108	[1105, 1112]	819	[817, 822]
phi.1	378	[377, 379]	68	[67, 69]
tau.1	419	[417, 421]	444	[442, 446]
tau.2	271	[269, 273]	294	[290, 298]
tau.3	446	[444, 449]	351	[348, 353]
tau.4	327	[325, 329]	389	[386, 390]
tau.5	333	[330, 334]	312	[312, 313]
tau.6	363	[361, 365]	325	[325, 326]
tau.7	431	[429, 433]	440	[437, 441]
tau.8	279	[277, 281]	387	[386, 390]
tau.9	372	[370, 375]	252	[251, 253]
tau.10	NA	NA	NA	NA
tau.11	NA	NA	NA	NA
tau.12	NA	NA	NA	NA
tau.13	NA	NA	NA	NA
mu.tau	357	[319, 398]	378	[356, 384]
sigma.tau	57	[43, 76]	48	[41, 62]
rho	0.00026	[0.00025, 0.00026]	0.00018	[0.00018, 0.00018]
delta	81.2	[77.7, 84.4]	140.5	[138.4, 142.6]
z	24	[2, 39]	2	[1, 8]
n	93.57	[93.04, 93.97]	51.8	[51.03, 51.97]
sMAE	0.0437		0.0357	

param	Estimate_us1_ili	CrI_us1_ili	Estimate_us9_ili	CrI_us9_ili
beta	0.5229	[0.5173, 0.5284]	0.4625	[0.451, 0.4731]
a	0.2021	[0.1909, 0.2182]	0.0613	[0.0594, 0.0627]
$gamma^{-1}$	2007	[1999, 2026]	1976	[1938, 2021]
phi.1	32	[30, 34]	1	[1, 5]
tau.1	317	[314, 318]	302	[297, 305]
tau.2	392	[390, 395]	399	[396, 403]
tau.3	278	[275, 281]	335	[331, 338]
tau.4	358	[355, 362]	350	[345, 356]
tau.5	357	[351, 362]	364	[361, 367]
tau.6	424	[418, 430]	387	[383, 392]
tau.7	367	[365, 369]	360	[357, 364]
tau.8	360	[357, 363]	362	[359, 364]
tau.9	379	[374, 383]	356	[352, 360]
tau.10	NA	NA	NA	NA
tau.11	NA	NA	NA	NA
tau.12	NA	NA	NA	NA
tau.13	NA	NA	NA	NA
mu.tau	360	[330, 394]	358	[333, 382]
sigma.tau	46	[30, 81]	32	[20, 60]
rho	0.00045	[0.00044, 0.00045]	0.00049	[0.00049, 0.0005]
delta	56.7	[52.5, 61.3]	179.9	[178.5, 180]
z	6	[3, 45]	1	[1, 8]
n	59.58	[58.15, 59.93]	67.57	[30.61, 69.3]
sMAE	0.0504		0.0606	
