# Supplementary material for: Repeatability and timing of tropical influenza epidemics
Source: PLoS Comput Biol. 2023 Jul 19;19(7):e1011317. doi: 10.1371/journal.pcbi.1011317 (PMC10389745; doi:10.1371/journal.pcbi.1011317)
Supplement: Attachment — Submitted filename: Repeatability and timing of tropical influenza epidemics.pdf [file pcbi.1011317.s009.pdf]

Review of the article "Repeatability and timing of tropical influenza epidemics" by J. L. Servadio, P. Q. Thai, M. Choisy, and M.F.Boni

This manuscript aims to characterize temporal patterns of influenza epidemics in Vietnam, specifically the variance in the timing of influenza outbreaks. The authors have collected data on the influenza virus in Vietnam, two regions of the USA, from 2006 to 2015, and the Netherlands and Denmark from 2010 to 2019. The authors then use MCMC to estimate the parameters of a mechanistic model. They design the term repeatability, which relates to the variance in the outbreak timing.

The purpose of the study is well-defined, and the text is clear overall. However, the model's section needs substantial improvement, especially on some specific hypotheses (please see my comments below). It also needs to be clarified how the authors quantify the standard deviations (SDs) to conclude whether repeatability exists. A significant example is the plot in Fig4 showing similar SDs levels for some regions in Vietnam compared to temperate regions. Still, the authors claim that *"influenza patterns are not repeatable or seasonal in Vietnam* ."A major concern is thus the need for precise characterization of repeatability and the several modeling assumptions without justification.

Here is a list of questions and comments I would like to see addressed in a revised submission.

<u>Major revisions and concerns about the methodology</u>

- Lines 162-178. Could the authors explain the need to detrend ILI cases in the specific way they did in this manuscript? I understand that some transformation might be beneficial to remove the effects of trends, but why is their zeta-score approach preferable in this case?

- One aspect of particular concern is the use of future data in the normalization process, given that each weekly case count is divided by the year-weekly average *centered* on the given week. Could the authors elaborate more on the preprocessing of epidemiological data, highlighting the benefits and limitations of your choice and adding references if needed?

- Lines 239-247. The inclusion of four recovery classes needs further explanation. Only a brief and vague sentence is added with reference to a paper on COVID-19 paper that seems unrelated to the present study. What are R_1,2,3,4? Why is only R_4 in dS/dt? What is the description of "v" in line 216?

- Lines 241 - 243. *In order to facilitate time periods where zero prevalence occurs, a model behavior was added outside of the differential equations where ILI+ is set to zero based on a parameter z (fit from the data) at which point all infected individuals immediately recover. When ILI+ falls below z, ILI+ is set to zero.*

- The sentence is confusing, and it is not clear why the authors assume that at some point, all infected individuals immediately recover. Further explanation is needed.

- Line 345. Some model fits for Vietnam are poor, especially for ILI+ data. The authors based their conclusions about the lack of repeatability of influenza epidemics in Vietnam on those plots. I don't see any table/discussion on the goodness of fit. I also wonder if aggregated monthly data would generate better fits. The authors could add some comments on the quality (noise) of the weekly data and limitations in the fitting process.

- Line 364 (Fig 4). The standard deviations for influenza A and B in central and southern Vietnam seem comparable to the ones from temperate regions. If repeatability is associated with lower variance, it appears that this result does not support the manuscript's central claim that *"influenza patterns are not repeatable or seasonal in Vietnam"*. The criteria for repeatable/seasonal influenza should be better explained.

  The authors do not show results for A/H1, A/H3, A, and B in temperate regions, which makes the comparison difficult. Please provide comparable results or explain in the results section why the subtype analysis was not performed in temperate regions.

<u>Minor revisions</u>

- Line 32. In the abstract, the authors could be more precise and explain how the variance of timings of peaks characterize repeatability. Is there a threshold in variance leading to a binary classification of repeatable vs. non-repeatable patterns? Adding SD values is not informative if not accompanied by a precise definition.

- Line 42. *"Much of the world experiences influenza in yearly recurring seasons, particularly in temperate locations. In temperate locations particularly, these seasons."*

- The expression "particularly in temperate locations" appears twice. Consider removing one of them.

- Line 123. *Here, we define "repeatability" as seasonality that occurs at a consistent time of year.*
- This definition is very important and should be added to the abstract.
- It is unclear how many subtypes of influenza virus are considered in the study. In lines 151 and 169, three subtypes are mentioned. But in line 177, four subtypes are mentioned. Also, in line 240, "Four recovery classes were chosen," is this because of four subtypes?
- In lines 244-247, an immigration parameter was added to avoid extinction in the model. What about the cases which are not reported and are still in the population? How does the model deal with that issue? I believe this immigration parameter is also considered as the cases which are not reported.
- Line 251. *To fit the model to observed data, we ran the model for a ten-year period as burn-in followed by a second ten-year period for model fitting and comparison to observed data.*
- A non-specialized reader may find the term "burn-in" obscure. The authors could add a short sentence explaining the jargon and why the procedure is necessary for their fitting process.
- Line 265. Please add references for MCMC sampling with the Metropolis-Hastings algorithm.
- Line 271. "We assumed that parameters…"
- Line 292. Add a reference for the Gelman-Rubin statistics.
- Line 324. Adding descriptive indicators of seasonality before the model fitting results is appropriate. I miss some exemplary figures in the main text, though. The SI presents a series of ACF and Wavelet plots. The authors could move some of those figures to the main text to highlight the dramatic differences in the results between Vietnam vs. the US or other temperate regions.
- Please double-check the manuscript for typos. For example, in line 29, "and then fit the model" should be changed to "and then fitted the model."
