# Supplementary material for: Repeatability and timing of tropical influenza epidemics
Source: PLoS Comput Biol. 2023 Jul 19;19(7):e1011317. doi: 10.1371/journal.pcbi.1011317 (PMC10389745; doi:10.1371/journal.pcbi.1011317)
Supplement: Attachment — Submitted filename: Servadio_response_to_reviewers_20230511.pdf [file pcbi.1011317.s010.pdf]

We thank the reviewers for their time and feedback; we were pleased to see the overall positive interest in our study, and our work has improved greatly as a result of the reviewers' comments. Below are our responses to the reviewers' individual comments. Each response appears in blue text, indented, underneath the pertinent comment. The most notable changes include expanded explanations and justifications of our analytic methods as well as subsequent analyses aiming to show that the 2009-2010 influenza pandemic does not influence our results. We also improved the presentations of some figures in order to more explicitly present our findings and support our conclusions. In making the appropriate revisions, we restructured our discussion section to more coherently and concisely incorporate the reviewers' suggestions.

REVIEWER 1

The study examines repeatability of Influenza like illness in Vietnam by analyzing surveillance data collected by 10 hospitals across three different locations within the country. The authors provide a clear definition of 'repeatability' used in the analysis as having consistent and predictable outbreaks at the same time each year. The authors build a mathematical model of influenza incorporating seasonal dynamics and use Bayesian inference to fit the model to observed influenza time series data. The data from Vietnam, Netherlands, Denmark, and United States is used to access hypothesis of repeatability of influenza in tropical and temperate regions.

The model and the analysis presented adequately supports the conclusion drawn by the authors that the incidence of influenza in Vietnam provides only weak evidence of seasonality and highly variant repeatability.
I think the study highlights importance of accounting the differences in influenza transmission between temperate and tropical regions instead of using a 'one size fits all' approach.

I have included some minor changes I'd like to see below:

Suggested changes and typo:

1. Line 31: Change 'norther' to northern

    This has been changed.

2. Line 162-178: Was detrending performed for all influenza subtype data or just for combined ILI+ data from Vietnam? Change the language to make this clear.

    Detrending was done for all individual (sub)types in addition to all ILI in Vietnam. This was also done for the data from four temperate locations. As a result, all 19 models (for A/H1, A/H3, A, B, ILI among each of northern, central, and southern Vietnam, plus four temperate locations) were fitted to

detrended data. This is now more explicitly stated in the text, appearing on lines 178-179 and 204-205 of the revised manuscript.

3. Line 420-422: "*The estimates for duration of immunity after infection for ILI+ and influenza B in Vietnam were approximately half that from the other model fits*". This seems like an interesting and possibly significant point. Can you state possible cause for this?

This is an interesting point, which led us to consider multiple facets of our analyses. First, we considered that it may be more appropriate to report modes of the posterior distributions in the results rather than medians. Thus, in the new revised analyses, all estimates are presented as modes from the posterior distributions. There were few places where this change was important, shown by the difference between the red dots (representing modes) and grey dots (representing medians) in Figure S3. It is also important to note that many of the estimated durations of immunity have low precision, as noted in Table S2.

Second, the duration of immunity parameter in mathematical models means something different depending on whether the incidence curve in the model is being fit to influenza A/H1N1, all ILI+, or just simply ILI. If we only consider a fit for a single subtype, then the duration of immunity estimate γ corresponds to immunity conferred by that subtype and for that subtype, but in the process we are ignoring the fact that infections are occurring with other subtypes. So, γ is overestimated here.

However, if we fit a model to ILI+ data (for all three subtypes), we are assuming "loss of immunity" when an individual is infected with a different type/subtype, but the reality is that this individual is likely still immune to their original infecting subtype (e.g. H1) but not to H3 or B which they got infected with recently. So, γ is underestimated here.

The challenge here is that we have not estimated the cross-immunity parameters (which would allow us to correct for the under/overestimation in these analyses). The best analysis done on this topic so far is by Yang et al (PLoS Comput Biol, 16:e1007989, 2020) where cross-immunity parameters are estimated, and they infer 2-3 years of immunity with posterior ranges that span 1.8 to 4 years. Our estimates are from 1.5 to 6 years.

Finally, we must note that we are fitting an <u>inherently unitless quantity</u> in this analysis (as are Yang et al in their analysis from 2020). In all flu model-fitting analyses, including ours, it is impossible to infer the absolute population numbers of infections every season (unless serology data are included or results from a health-care utilization study are available, but this is never the case). Thus, none of the model fits, including the Denmark, Netherlands, and US model fits, are able to confidently infer <u>how many</u>

individuals become infected in each epidemic. This estimate of absolute attack rate is the most important factor in determining what the 'immune waning' rate is. If 25% of individuals are expected to be infected every year in a stable seasonal flu model, then the waning rate in this system should be inferred at about four years. Unless a data-fitting analysis like this includes some information on 'total population size infected' it is clear that we can have confidence in the waning estimates.

These matters motivate much future work. We have incorporated these topics into our main text to discuss how our interpretations are limited by the model form and constraints of our analyses. This is discussed on lines 615-637 of the revised manuscript.

4. Suggestion: The authors have used data from three temperate region in addition to a tropical region Vietnam. Why was this choice made rather than say using data from another tropical region. Is this mainly due to the availability of data on influenza outbreaks? In particular, I think the statement about generality made in the abstract (line 37: "*This suggests that influenza patterns are not repeatable or seasonal in Vietnam. Influenza prevention in Vietnam, and by extension other tropical locations, therefore cannot rely anticipating regularly occurring outbreaks.")* would be strengthened by inclusion of data from another tropical region. This would rule out the conclusion of unpredictable seasonality of influenza incidences in tropics from being specific to a single country. Not entirely necessary to conduct more analysis since that might be significantly more work but maybe the statement in the abstract can be revised.

The data from temperate locations were used to draw comparisons between Vietnam and locations with more temperate climates, where seasonality is well defined. This was to show that our model is capable of detecting seasonality when it does exist. The three countries used had the most complete data from their respective sources. While these sources only have incidence data for all ILI, without specific subtype information, they still can serve as comparisons.

While some other publicly available data sets exist for influenza burden in other tropical locations, they are often based on passive surveillance and not separated by (sub)type the way our data from the sentinel surveillance system in Vietnam are. One exception is a dataset from sentinel surveillance in Hong Kong used by Yang et al. (2020, PLoS Comp Biol), but inclusion of one additional data set, while additional evidence, is still not sufficient for generalizing to all of the tropics.

The point about generalizing to tropical world regions based on findings from Vietnam is very important. We recognize that it is challenging to generalize to the tropics when using data from only one country. To avoid overstepping, we have removed this from the abstract, and in the main text

made it clear that we are only acknowledging the possibility that our lack of repeatability in Vietnam may apply elsewhere rather than stating with confidence that this is true.

Additional comment: The GitHub page provided for data and code did not exist at the time of this review. Please change that.

Thank you for catching this oversight. The github repository has now been made public.

REVIEWER 2

This manuscript aims to characterize temporal patterns of influenza epidemics in Vietnam, specifically the variance in the timing of influenza outbreaks. The authors have collected data on the influenza virus in Vietnam, two regions of the USA, from 2006 to 2015, and the Netherlands and Denmark from 2010 to 2019. The authors then use MCMC to estimate the parameters of a mechanistic model. They design the term repeatability, which relates to the variance in the outbreak timing. The purpose of the study is well-defined, and the text is clear overall. However, the model's section needs substantial improvement, especially on some specific hypotheses (please see my comments below). It also needs to be clarified how the authors quantify the standard deviations (SDs) to conclude whether repeatability exists. A significant example is the plot in Fig4 showing similar SDs levels for some regions in Vietnam compared to temperate regions. Still, the authors claim that *"influenza patterns are not repeatable or seasonal in Vietnam* ."A major concern is thus the need for precise characterization of repeatability and the several modeling assumptions without justification. Here is a list of questions and comments I would like to see addressed in a revised submission.

Major revisions and concerns about the methodology

- Lines 162-178. Could the authors explain the need to detrend ILI cases in the specific way they did in this manuscript? I understand that some transformation might be beneficial to remove the effects of trends, but why is their zeta-score approach preferable in this case?

This is an important and underappreciated thought regarding our analyses. First, the detrending needed to be done in a way that preserved the ratio of consecutive incidence data points. In other words, in the final detrended time series, y_t and y_{t+1} (incidence data on consecutive weeks) need to have their ratio y_t / y_{t+1} preserved as closely as possible as this ratio is the direct mechanistic result of a pathogen (with approximately a 1-week serial interval) spreading in a population. As an example, a z-score approach would not work here since z-scores can be negative.

Second, the detrending window needs to be 365 days long to preserve any annual signals in the data. Since the main question in this analysis focused on whether annual signals or repeatable annual signals existed in the data, it is important that the detrending process not erase any annual signals. So, if points a and b are exactly 365 days apart in the data, and if there truly is annual forcing in the system, then a and b should have the same relationship to their neighboring points. In other words, if a is a peak, b should be a peak. If a is a trough, then b should also be a trough. By using a 365-day window, we ensure that a and b have completely separate smoothing windows, and the detrending of a does not affect the detrending of b (nor vice versa).

Essentially, if aiming to preserve (1) annual signals and (2) mechanistic autocorrelation due to pathogen transmission, one needs to have a 365-day window to preserve (1) and some approach (like our ζ-score approach) to preserve (2).

We have elaborated our explanation of our detrending methods and motivations, which can be found on lines 163-176 of the revised manuscript.

● One aspect of particular concern is the use of future data in the normalization process, given that each weekly case count is divided by the year-weekly average *centered* on the given week. Could the authors elaborate more on the preprocessing of epidemiological data, highlighting the benefits and limitations of your choice and adding references if needed?

The incorporation of future data would be problematic if we were forecasting. However, in our work, we are simply trying to transform data sets to make them comparable for a model fit. The essential weekly incidence unit we are using is "x% above or below some yearly mean" and the yearly means (standard moving averages) are computed as all data 6 months after and 6 months before the focal data point. This is a standard detrending approach in epidemiology.

This detrending method works well in our analysis because it successfully filters out long-term trends that may result from inconsistencies in data collection and reporting among the sentinel clinics during the period of observation, and it allows us to make weighted sums of ζ-scores from multiple sites.

This explanation has been added to the description of the data detrending, seen on lines 163-176 of the revised manuscript.

● Lines 239-247. The inclusion of four recovery classes needs further explanation. Only a brief and vague sentence is added with reference to a paper on COVID-19 paper that seems unrelated to the present study. What are R_1,2,3,4? Why is only R_4 in dS/dt? What is the description of "v" in line 216?

> Multiple recovery classes were chosen to prevent rapid loss of immunity following infection. Gamma distributed immune durations are considered more realistic compared to Exponentially distributed, and including multiple recovery classes allows this as the sum of Exponentially distributed rates is Gamma.
>
> Given the purpose of these multiple recovery classes as a sequence, only R_4 is present in dS/dt because immunity loss occurs only from R_4. Infected populations need to move across R_1 through R_4 before losing immunity. The distinction of four recovery classes as a sequence, an additional, better citation, and clearer statement for the motivation to use four recovery classes can be found on lines 255-259 of the revised manuscript.
>
> The parameter nu in the equation is the rate at which infected individuals recover. We assume a 5-day duration of infection.

● Lines 241 - 243. *In order to facilitate time periods where zero prevalence occurs, a model behavior was added outside of the differential equations where ILI+ is set to zero based on a parameter z (fit from the data) at which point all infected individuals immediately recover. When ILI+ falls below z, ILI+ is set to zero.*
The sentence is confusing, and it is not clear why the authors assume that at some point, all infected individuals immediately recover. Further explanation is needed.

> We agree that the text benefits from additional detail. The purpose of this parameter was to prevent a situation where an epidemic is ending, but a very small lingering infected population continues to infect the susceptible population and prolong the epidemic. The purpose of this was to fit the observed behavior in the data where, particularly for individual (sub)types, periods of zero incidence occur. Additional detail has been added to this paragraph for clarity, as seen on lines 259-266 of the revised manuscript.

● Line 345. Some model fits for Vietnam are poor, especially for ILI+ data. The authors based their conclusions about the lack of repeatability of influenza epidemics in Vietnam on those plots. I don't see any table/discussion on the goodness of fit. I also wonder if aggregated monthly data would generate better fits. The authors could add some comments on the quality (noise) of the weekly data and limitations in the fitting process.

> It is true that the fits particularly for ILI+ in Vietnam are much poorer than those for the individual A subtypes or for the temperate locations. In order to

find a goodness of fit method that can compare similar models fitted to different data sets, we calculated the mean absolute error, standardized by the maximum observed value in each data set, for each parameter set from the posterior distributions and the observed data. The average of these has been added to Table S2 to quantify how much closer the models for A/H1 and A/H3 can match the data compared to combined ILI+ in Vietnam. In addition to adding this to Table S2, this is reported in the text on lines 380-385 and 439-441 of the revised manuscript. The fact that not all model fits are close, precise fits to data is also noted in the discussion. This is a limitation of our model, which is designed for single pathogens. This discussion is seen on lines 600-609 of the revised manuscript.

In light of this, we have also changed the presentation of Figure 4 (previously Figure 3) to distinguish that, in the case of Vietnam, where (sub)types of influenza are not synchronized, a single-strain SIRS model is unlikely to provide a good fit to data containing multiple subtypes. This will clarify our finding that our model is better suited for single-subtype fits rather than fits for combined ILI+.

● Line 364 (Fig 4). The standard deviations for influenza A and B in central and southern Vietnam seem comparable to the ones from temperate regions. If repeatability is associated with lower variance, it appears that this result does not support the manuscript's central claim that *"influenza patterns are not repeatable or seasonal in Vietnam"* . The criteria for repeatable/seasonal influenza should be better explained. The authors do not show results for A/H1, A/H3, A, and B in temperate regions, which makes the comparison difficult. Please provide comparable results or explain in the results section why the subtype analysis was not performed in temperate regions.

Individual subtype data were not available for our temperate regions, so we are only able to fit the model to data from all ILI+ in temperate locations. We have added to the discussion to mention that this presents the challenge of having well-fit models for ILI+ in temperate locations, well-fit models for influenza subtypes in Vietnam, and poorly-fit models for ILI+ in Vietnam. This can be found on lines 644-653 of the revised manuscript.

To address the fact that there is overlap in the credible intervals for the standard deviation between certain influenza fits in Vietnam and the fits for temperate locations, we have changed the error bars in the plots to violin plots to better show the extent of the overlap in the credible intervals. It is also worth noting that, among 15 model fits for Vietnam, lower standard deviation of timing between epidemics only is found in three to four of the fits. In addition, based on a different comment, we ran additional samples for some of the models to be more confident in convergence and the representativeness of the posterior distributions. The text now acknowledges these fits from Vietnam with low standard deviations for peak

timings, but clarifies that the overall trend across the model fits shows much weaker evidence of repeatability in Vietnam. This is discussed on lines 598-609 of the revised manuscript.

We are not able to create a cutoff to classify repeatability into a binary indicator; doing so requires the use of an arbitrary cutoff. This fact, and the fact that we did not set a cutoff a priori, would render such a cutoff meaningless. Throughout the manuscript, we describe repeatability in this qualitative manner as a result. We mention this limitation on lines 514-516 of the revised manuscript.

Minor revisions

● Line 32. In the abstract, the authors could be more precise and explain how the variance of timings of peaks characterize repeatability. Is there a threshold in variance leading to a binary classification of repeatable vs. non-repeatable patterns? Adding SD values is not informative if not accompanied by a precise definition.

Similar to the previous comment, we do not have an arbitrary cutoff designating a binary classification of repeatability. Instead, the comparison between the SD fits for Vietnam and for temperate locations, where seasonality is well-defined, provides our evidence (or lack thereof) of repeatability. We have emphasized in the abstract that the observed differences in SD provide qualitative evidence of seasonality rather than exceeding a cutoff.

● Line 42. *"Much of the world experiences influenza in yearly recurring seasons, particularly in temperate locations. In temperate locations particularly, these seasons."*
The expression "particularly in temperate locations" appears twice. Consider removing one of them.

This has been corrected to avoid using the phrase twice.

● Line 123. *Here, we define "repeatability" as seasonality that occurs at a consistent time of year.*
This definition is very important and should be added to the abstract.

This has been added to the abstract. The added phrase can be seen on lines 28-29 of the revised manuscript.

● It is unclear how many subtypes of influenza virus are considered in the study. In lines 151 and 169, three subtypes are mentioned. But in line 177, four subtypes are mentioned. Also, in line 240, "Four recovery classes were chosen," is this because of four subtypes?

> We appreciate the need for clarification here. PCR testing indicated three subtypes (A/H1, A/H3, and B), and we considered these three as well as type A (sum of A/H1 and A/H3) and combined ILI+ (sum of A/H1, A/H3, and B). This leads to five different analyses based on flu subtypes and combinations of them. This is now stated more explicitly, as seen on lines 187-190 of the revised manuscript.
>
> The four recovery classes are unrelated to the number of subtypes. Based on a previous comment, we elaborated the purpose of the multiple recovery classes, which was to reduce variance in the duration of immunity within the population and prevent instantaneous loss of immunity. This elaboration is noted on lines 255-260 of the revised manuscript.

● In lines 244-247, an immigration parameter was added to avoid extinction in the model. What about the cases which are not reported and are still in the population? How does the model deal with that issue? I believe this immigration parameter is also considered as the cases which are not reported.

> While the immigration parameter does represent any type of transmission that occurs outside of the model system, cases that occur but are not reported are primarily handled in our model through a reporting fraction. This scales down modeled incidence based on what fraction of cases are detected by the surveillance system. We estimated this parameter through our model fitting. However, this parameter cannot be interpreted only as the fraction of cases that are detected and reported because it is used to scale down modeled case counts within the model system to a unitless value to compare to detrended data. This has been added to the paragraph describing parameters in the model (lines 269-271 and 281-283 of the revised manuscript).

● Line 251. *To fit the model to observed data, we ran the model for a ten-year period as burn-in followed by a second ten-year period for model fitting and comparison to observed data.* A non-specialized reader may find the term "burn-in" obscure. The authors could add a short sentence explaining the jargon and why the procedure is necessary for their fitting process.

> This added explanation is advantageous for both non-specialized readers as well as readers who know burn-in primarily in other contexts such as MCMC. The fact that we used MCMC with a burn-in period heightens this risk of

confusion. We have added a sentence describing the burn-in period in this context (lines 276-280 of the revised manuscript).

● Line 265. Please add references for MCMC sampling with the Metropolis-Hastings algorithm.

Appropriate references have been added. The citations appear on line 293 of the revised manuscript

● Line 271. "We assumed that parameters…"

We reworded this sentence for clarity. It can be found on lines 299-300 of the revised manuscript.

● Line 292. Add a reference for the Gelman-Rubin statistics.

An appropriate reference has been added. The citation can be found on line 320 of the revised manuscript.

● Line 324. Adding descriptive indicators of seasonality before the model fitting results is appropriate. I miss some exemplary figures in the main text, though. The SI presents a series of ACF and Wavelet plots. The authors could move some of those figures to the main text to highlight the dramatic differences in the results between Vietnam vs. the US or other temperate regions.

In order to allow better comparisons in the main text, we have moved parts of the Figures S1 and S2 into the main text; they comprise Figure 3 in the revised manuscript. Including the ACF and wavelet visualizations for southern Vietnam and the four temperate locations allows the differences described in the text to be seen visually while remaining concise. Figures S1 and S2 remain to show the full results.

● Please double-check the manuscript for typos. For example, in line 29, "and then fit the model" should be changed to "and then fitted the model."

We corrected the “fitted” indicated in the text (and in other locations throughout) and checked the full text for other typos or grammatical errors.

REVIEWER 3

with pleasure I read your paper. I have a number of questions in particular related to the methodology:

- you compare sentinel hospital data from Vietnam with surveillance data from the Netherlands Denmark and the USA.
It is unclear where the data from the Netherlands Denmark and USA do come from? Hospital sector, primary care or community? The current presentation of data looks like a comparison between apples and pears.

> Additional information regarding the data collection in Netherlands, Denmark, and United States have been added to the main text. These can be found on lines 192-197 and 200-201 of the revised manuscript.
>
> It is true that, because the data in these countries were not collected in the same manner as the data in Vietnam, there will be inherent differences in the data and what they represent. We have added text in the discussion to acknowledge this limitation. It can be seen in lines 644-653 of the revised manuscript.

- you include the pandemic season 2009-10 in your analysis: could you run a analysis without those years to see whether this has an effect on the results. It is known from literature that the 09 pandemic had impact on the next flu seasons in many countries.

> In order to show whether omitting the 2009-2010 H1N1 pandemic affects our results, we reran selected models. Because our fits for subtypes A/H1 and A/H3 were the closest fits in northern, central, and southern Vietnam, we refitted the models for those two subtypes in all three locations in Vietnam. Because our data from the United States also include the timing of this pandemic, we also reran the two fits from the US.
>
> We conducted this refitting omitting the pandemic in three different ways: (1) based on the WHO start and end dates June 2009 – Aug 2010), (2) based on the start of the pandemic's effect on Vietnam (July 2009) and lasting 18 months, and (3) based on the start of the pandemic's effect on Vietnam (July 2009) and lasting 36 months.
>
> The results from these additional fits show that the variability in peak timing was remained higher in Vietnam compared to the United States. During the periods of omitted data, epidemics were able to occur at various points. This is not surprising given the absence of data to compare to the peaks during this time. This also can allow timings to occur at points that may align with repeatable patterns, but the standard deviations for timings of epidemic peaks remained higher, as seen in Figure S4-S5 of the revised manuscript.

These results are also reported in the main text, on lines 475-484 of the revised manuscript.

Furthermore I am not convinced about the way you present your figure 3 > I do understand the results from Vietnam but the comparison with the ILI figures from NL DK and USA next to the Vietnamese figures confuses. What do the authors want to compare in this figure? Please clarify this also in the table text

The purpose of this figure was to visually show the fits for the individual models. The plotted fits from temperate locations are not meant to append the rows of the fits from Vietnam. In order to make this distinction clearer, we have separated the fits from temperate locations from the fits from Vietnam. To further illustrate how the fits for individual subtypes of influenza in Vietnam showed better model fits, we also separated the fits of individual subtypes from the fits of combinations of subtypes, which showed poorer fits. This figure is now Figure 4 in the revised manuscript.

The introduction and discussion are fine

Thank you. You will find that, particularly in the discussion, additional points have been added that discuss notable findings and interpretations of our work that were given less emphasis in the original manuscript.
