# Supplementary material for: Repeatability and timing of tropical influenza epidemics
Source: PLoS Comput Biol. 2023 Jul 19;19(7):e1011317. doi: 10.1371/journal.pcbi.1011317 (PMC10389745; doi:10.1371/journal.pcbi.1011317)
Supplement: Attachment — Submitted filename: plosCompBio.docx [file pcbi.1011317.s008.docx]

The study examines repeatability of Influenza like illness in Vietnam by analyzing surveillance data collected by 10 hospitals across three different locations within the country. The authors provide a clear definition of ‘repeatability’ used in the analysis as having consistent and predictable outbreaks at the same time each year. The authors build a mathematical model of influenza incorporating seasonal dynamics and use Bayesian inference to fit the model to observed influenza time series data. The data from Vietnam, Netherlands, Denmark, and United States is used to access hypothesis of repeatability of influenza in tropical and temperate regions.

The model and the analysis presented adequately supports the conclusion drawn by the authors that the incidence of influenza in Vietnam provides only weak evidence of seasonality and highly variant repeatability.

I think the study highlights importance of accounting the differences in influenza transmission between temperate and tropical regions instead of using a ‘one size fits all’ approach.

I have included some minor changes I’d like to see below:

**Suggested changes and typo:**

1. Line 31: Change ‘norther’ to northern
2. Line 162-178: Was detrending performed for all influenza subtype data or just for combined ILI+ data from Vietnam? Change the language to make this clear.
3. Line 420-422: “*The estimates for duration of immunity after infection for ILI+ and influenza B in Vietnam were approximately half that from the other model fits*”. This seems like an interesting and possibly significant point. Can you state possible cause for this?
4. Suggestion: The authors have used data from three temperate region in addition to a tropical region Vietnam. Why was this choice made rather than say using data from another tropical region. Is this mainly due to the availability of data on influenza outbreaks? In particular, I think the statement about generality made in the abstract (line 37: “*This suggests that influenza patterns are not repeatable or seasonal in Vietnam. Influenza prevention in Vietnam, and by extension other tropical locations, therefore cannot rely anticipating regularly occurring outbreaks.”)* would be strengthened by inclusion of data from another tropical region. This would rule out the conclusion of unpredictable seasonality of influenza incidences in tropics from being specific to a single country. Not entirely necessary to conduct more analysis since that might be significantly more work but maybe the statement in the abstract can be revised.

**Additional comment:** The GitHub page provided for data and code did not exist at the time of this review. Please change that.
